# The Dual Role of the NFATc2/galectin‐9 Axis in Modulating Tumor‐Initiating Cell Phenotypes and Immune Suppression in Lung Adenocarcinoma

**DOI:** 10.1002/advs.202306059

**Published:** 2024-03-25

**Authors:** Zhi‐Jie Xiao, Si‐Qi Wang, Jun‐Jiang Chen, Yun Li, Yuchen Jiang, Vicky Pui‐Chi Tin, Jia Liu, Huiyi Hu, Maria Pik Wong, Yihang Pan, Judy Wai Ping Yam

**Affiliations:** ^1^ Scientific Research Centre The Seventh Affiliated Hospital Sun Yat‐sen University Shenzhen 518000 China; ^2^ Shenzhen Key Laboratory of Bone Tissue Repair and Translational Research The Seventh Affiliated Hospital Sun Yat‐sen University Shenzhen 518000 China; ^3^ Department of Pathology School of Clinical Medicine The University of Hong Kong Hong Kong 999077 Hong Kong; ^4^ State Key Laboratory of Stem Cell and Reproductive Biology Institute of Zoology Chinese Academy of Sciences Beijing 100101 China; ^5^ Key Laboratory of Organ Regeneration and Reconstruction Chinese Academy of Sciences Beijing 100101 China; ^6^ Beijing Institute for Stem Cell and Regenerative Medicine Beijing 100101 China; ^7^ Department of Physiology School of Medicine Jinan University Guangzhou 510000 China; ^8^ Department of Thoracic Surgery The Seventh Affiliated Hospital of Sun Yat‐Sen University Shenzhen China

**Keywords:** NFATc2, galectin‐9, immune, lung adenocarcinoma, tumor‐initiating cells

## Abstract

Tumor‐initiating cells (TICs) resilience and an immunosuppressive microenvironment are aggressive oncogenic phenotypes that contribute to unsatisfactory long‐term outcomes in lung adenocarcinoma (LUAD) patients. The molecular mechanisms mediating the interaction between TICs and immune tolerance have not been elucidated. The role of Galectin‐9 in oncogenesis and immunosuppressive microenvironment is still unknown. This study explored the potential role of galectin‐9 in TIC regulation and immune modulation in LUAD. The results show that galectin‐9 supports TIC properties in LUAD. Co‐culture of patient‐derived organoids and matched peripheral blood mononuclear cells showed that tumor‐secreted galectin‐9 suppressed T cell cytotoxicity and induced regulatory T cells (Tregs). Clinically, galectin‐9 is upregulated in human LUAD. High expression of galectin‐9 predicted poor recurrence‐free survival and correlated with high levels of Treg infiltration. *LGALS9*, the gene encoding galectin‐9, is found to be transcriptionally regulated by the nuclear factor of activated T cells 2 (NFATc2), a previously reported TIC regulator, via in silico prediction and luciferase reporter assays. Overall, the results suggest that the NFATc2/galectin‐9 axis plays a dual role in TIC regulation and immune suppression.

## Graphical abstract

This study unveils the important role of NFATc2/galectin‐9 axis in modulating tumor‐initiating cell (TIC) phenotypes and immune suppression in lung cancer. Galectin‐9 secreted from tumor cells suppresses CD8^+^ T cell cytotoxicity and induces regulatory T cell fraction. The study illustrates galectin‐9 to be a promising prognostic marker and therapeutic target for targeting the TIC pool and immune‐suppressive microenvironment.

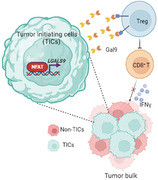

## 1 Introduction

Lung cancer is the most lethal malignancy and accounts for 18% of all cancer deaths worldwide.^[^ ^1^ ^]^ Despite the introduction of targeted therapy, treatment failure in advanced cancers is common, and new therapeutic approaches are needed. The acquisition of drug resistance and aggressive growth properties is thought to arise from the selection of a group of cells that display enhanced motility, metastasis, resilience, and survival, etc., termed cancer stem cells or tumor initiation cells (TICs).^[^ ^2^, ^3^ ^]^ Targeting TICs through molecular mechanisms that sustain such resilience might be a plausible strategy for improving treatment outcomes.^[^ ^4^ ^]^

In recent years, insight into the interactions of these cells with the immune microenvironment has revealed the crucial vulnerability of cancer cells, which might be useful for developing new therapeutic approaches. Established cancers can induce immune tolerance and escape elimination through immune checkpoint engagement, affecting the regulatory T lymphocyte (Treg) surge and cytotoxic T cell suppression. Currently, successful targeting of the PDL1‐PD1 immune checkpoint in several clinical lung adenocarcinomas (LUADs) illustrates the feasibility of this approach, but the response rate is reported to be only ≈30%, suggesting that blockade of alternative or multiple immune checkpoints might be needed to benefit a broader range of patients.^[^ ^5^, ^6^, ^7^, ^8^ ^]^

Whether TICs, the conceptually most aggressive and resilient cell population in cancer, might also show augmented immune escape capacity compared to non‐TICs has been investigated. Indirect evidence from an earlier mouse model study showed that vaccine‐activated cytotoxic T lymphocytes could drive cancer evolution by selecting and enriching tumor cells displaying stem‐like phenotypes through Nanog expression.^[^ ^9^ ^]^ Indeed, recent studies have suggested that other mechanisms, such as decreased TIC immunogenicity, occur due to reduced antigen processing and presentation machinery.^[^ ^10^ ^]^ An immunosuppressive signature has also been associated with cancer stemness.^[^ ^11^, ^12^ ^]^ For example, TIC has been shown to attenuate immune surveillance by upregulating the immunosuppressive ligand PDL1 or recruiting immunosuppressive cells to the tumor microenvironment.^[^ ^13^, ^14^ ^]^ These findings suggest that targeting the immunomodulatory molecules of TICs might be a promising treatment strategy. However, in lung cancer, the role of TICs in immune modulation and the underlying mechanisms remain largely unknown.

Galectin‐9 is an intracellular and secreted glycoprotein involved in cell‐cell and cell‐matrix interactions between innate and adaptive immune cells. It is a ligand for the receptor T‐cell immunoglobulin and mucin domain containing‐3 (TIM‐3), the binding of which activates the immune checkpoint pathway, leading to apoptosis and proliferation arrest of cytotoxic T cells.^[^ ^15^ ^]^ However, the role of galectin‐9 in malignancies is controversial and apparently cancer‐dependent. We previously reported that high expression of the crucial immune cell factor nuclear factor of activated T cells 2 (NFATc2) promotes TIC phenotypes in LUAD.^[^ ^16^ ^]^ In silico prediction suggested that NFATc2 could be a transregulator of *LGALS9*, the gene encoding galectin‐9. These findings led to the hypothesis that galectin‐9 might be a target of NFATc2 that mediates immune escape of TICs in LUAD. However, the potential role of galectin‐9 in regulating stem‐like phenotypes is largely unknown and has been implicated in only one study of leukemia stem cells,^[^ ^17^ ^]^ while its effects on the TIC of solid tumors remain unknown. Specifically, whether galectin‐9 is involved in TIC regulation and immune modulation in LUAD has never been reported.

We cocultured newly raised LUAD or normal lung organoids and peripheral blood mononuclear cells (PBMCs) from the same patient and demonstrated that tumor‐secreted galectin‐9 induced the apoptosis of cytotoxic T cells and suppressed T cell proliferation by increasing Treg numbers. Considering its role in TICs, we demonstrated that galectin‐9 supported essential phenotypes, including self‐renewal, tumorigenesis, and resistance to both chemotherapy and targeted therapy. The results were also verified using established lung cancer cell lines and a syngeneic mouse model of lung cancer. In clinical lung cancer, we found that high expression of galectin‐9 in tumor cells predicted poor survival and was positively correlated with Treg infiltration. At the molecular level, galectin‐9 was verified to be a downstream target of NFATc2. Overall, our findings suggest that galectin‐9 plays a dual role in TIC regulation and immune suppression and suggest that it is a potential treatment target for eradicating tumor resilience and unleashing immune inhibition in LUAD.

## 2 Results

### 2.1 Galectin‐9 Enhanced the TIC Proportion, Cell Mobility, Cancer Drug Response and Tumorigenesis

**Figure 1:**
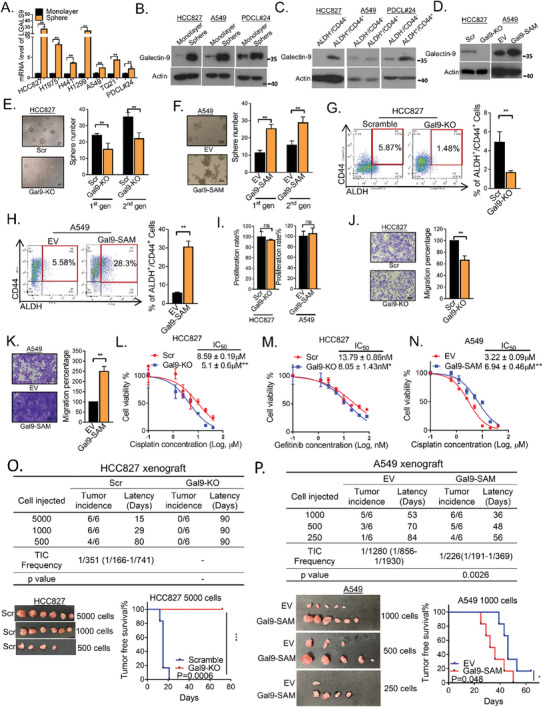
Galectin‐9 supported the TIC phenotypes of lung adenocarcinoma. A) Expression of *LGALS9* in tumor spheres and monolayers determined by qPCR. B) Expression of galectin‐9 in tumor spheres and monolayers determined by western blot. C) Expression of galectin‐9 and NFATc2 in the ALDH^+^/CD44^+^ and ALDH^−^/CD44^−^ subsets determined by western blot analysis. D) Expression of galectin‐9 in HCC827 and A549 cells with or without galectin‐9 knockout or overexpression determined by western blot. E,F) Sphere formation assay in HCC827 and A549 cells with galectin‐9 manipulation. G,H) ALDH/CD44 expression was analyzed by flow cytometry in HCC827 and A549 cells with galectin‐9 knockout or overexpression. I) BrdU proliferation assay of HCC827 and A549 cells after galectin‐9 manipulation. J,K) Migration assay of HCC827 and A549 cells with galectin‐9 manipulation. L,M) Effects of galectin‐9 knockout on cisplatin (L) and gefitinib (M) sensitivity in HCC827 cells determined by MTT assay. N) Effect of galectin‐9 overexpression on the cisplatin sensitivity of A549 cells, as determined by MTT assay. O,P) In vivo limiting dilution assay in which decreasing numbers of HCC827 (O) or A549 cells (P) were injected via a galectin‐9 mixture into SCID mice; *n* = 6 for each group. The tumor incidence, latency, and estimated TIC frequency calculated by L‐cal software are shown in the table (upper panel). Representative tumor images of the in vivo limiting dilution assay (lower left panel) and the effect of galectin‐9 manipulation on the tumor‐free survival of HCC827 or A549 xenografts analyzed by the log‐rank test are shown (lower right panel). ^*^ *p* < 0.05, ^**^ *p* < 0.01 versus the control according to Student's t‐test. The data are presented as the means±SDs of three replicates.

To investigate whether galectin‐9 was involved in maintaining tumor stemness, its expression was first compared between TICs and non‐TICs using 2 surrogate markers, namely, the ability of cells to form tumor spheres and high‐level coexpression of the lung cancer stem cell markers ALDH and CD44 (ALDH^+^/CD44^+^).^[^ ^18^ ^]^ The tumor spheres presented higher galectin‐9 levels at both the transcript (*LGALS9*) and protein levels than the monolayers (**Figure** **1**A,B). Similar results were observed for the ALDH^+^/CD44^+^ TIC subset compared to those for the non‐TIC subset, which had the lowest level of ALDH/CD44 coexpression (Figure 1C). To study whether galectin‐9 was functionally involved in supporting TICs, *LGALS9* knockout (Gal9‐KO) clones of HCC827 and H1975 cells were generated (Figure 1D), and the expression of galectin‐9 was further rescued by overexpressing *LGALS9* in H1975 cells (Figure S1A, Supporting Information). Gal9‐KO led to significantly suppressed sphere formation, but this effect was reversed by overexpressing galectin‐9 (Gal9‐KO/OE), as shown in H1975 cells (Figure 1E; Figure S1B, Supporting Information). Furthermore, for A549 cells, which exhibited relatively low de novo galectin‐9 levels (Figure S2D, Supporting Information), endogenous overexpression of galectin‐9 via the CRISPR‐based SAM system (Gal9‐SAM) led to enhanced sphere formation (Figure 1D,F). Galectin‐9 knockout in HCC827 cells also led to suppression of the ALDH^+^/CD44^+^ TIC proportion, while overexpressing galectin‐9 in A549 cells led to increased ALDH^+^/CD44^+^ TIC (Figure 1G,H). Furthermore, to clarify whether the change in sphere numbers was due to an altered cell proliferation state, a BrdU assay was used, which showed that the proliferation rate of HCC827, H1975, and A549 cells was not significantly changed by either galectin‐9 knockout or overexpression, suggesting that galectin‐9 mainly affects stemness rather than proliferation in the evaluated LUAD cells (Figure 1I; Figure S1C, Supporting Information). Next, the effects of galectin‐9 on tumor metastasis, cancer drug resistance, and tumorigenesis were evaluated. HCC827 and H1975 cells with galectin‐9 knock‐out showed suppressed capacities for cell migration, as well as sensitization to gefitinib and cisplatin treatment, while the rescue of galectin‐9 expression in H1975 reversed these inhibitory effects. Conversely, overexpression of galectin‐9 in A549 cells promoted migration and cisplatin resistance (Figure 1J–N; Figure S1D–F, Supporting Information). To demonstrate the in vivo actions of galectin‐9, tumorigenesis was assessed with limiting dilution assays. HCC827 cells with Gal9‐KO significantly reduced tumorigenesis, prolonged tumor‐free survival, and resulted in a failure of tumor engraftment with 5000 cells observed for 3 months (Figure 1O). In contrast, A549 cells with Gal9‐SAM had a significantly increased tumor incidence, higher TIC frequency, and shorter tumor‐free survival (Figure 1P). Overall, these results indicated that galectin‐9 plays functional roles in supporting the TIC phenotypes of LUAD.

### 2.2 Galectin‐9 Supported TIC Phenotypes in Patient‐Derived LUAD Organoids

**Figure 2:**
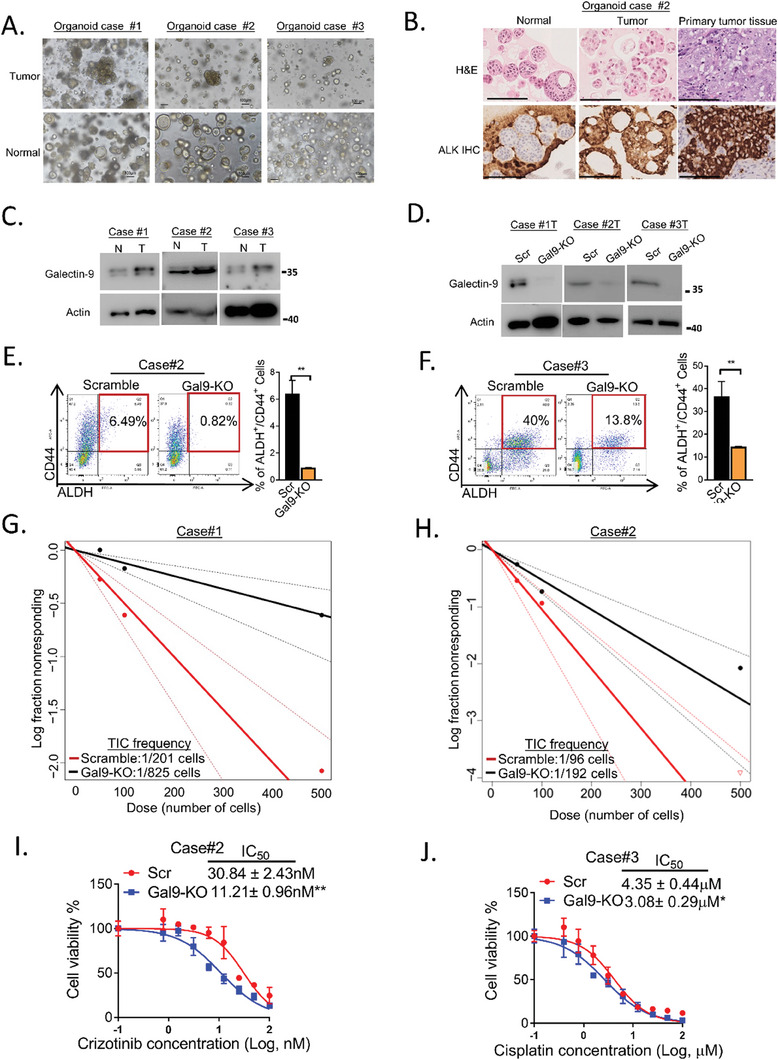
Galectin‐9 supported TIC phenotypes in LUAD organoids. A) Representative bright field images of paired normal and tumor organoids cultured from patient‐derived tissues. B) Representative H&E and IHC staining of ALK in tumor organoids and the corresponding primary tissue. Scale bar: 40 µM. C) Expression of galectin‐9 in normal lung organoids and LUAD organoids determined by western blot analysis. D) Expression of galectin‐9 in LUAD organoids with or without galectin‐9 knockout determined by western blot analysis. E–F) The ALDH/CD44 TIC fraction of tumor organoid Case #2 (E) and Case #3 (F) after galectin‐9 manipulation was analyzed by flow cytometry. G,H) An in vitro limiting dilution assay was performed. Tumor organoids from Case #1 (G) and Case #2 (H), with or without galectin‐9 KO, were digested into single cells. A total of 250, 50, and 5 cells per well were seeded into 32, 64, and 96 wells of 96‐well plates, respectively. The cells were cultured in TIC medium and allowed to form tumor spheres for 3 weeks. The number of wells with tumorspheres was counted for each cell dose. Limiting dilution analyses were performed using Extreme Limiting Dilution Analysis software. (http://bioinf.wehi.edu.au/software/elda) I–J) Cell viability assay of Crizotinib sensitivity in Case #2 (I) and cisplatin sensitivity in Case #3 (J) with or without galectin‐9 knockout using CellTiter‐Glo reagents. ^*^ *p *< 0.5, ^**^ *p* < 0.01 versus the control according to Student's t‐test. The data are presented as the means±SDs of three replicates.

Because cancer organoid models convey greater clinical relevance due to the conservation of genetic, histologic, and differentiation characteristics of their parental tumors,^[^ ^19^ ^]^ 3 pairs of patient‐derived LUADs and corresponding normal lung organoids were generated (Table S1, Supporting Information). The normal lung organoids were more uniform in size and had a rounded configuration and empty cores, while the tumor organoids were variably sized and had irregular outlines with solid cores (**Figure** **2**A). Tumor organoid #2 was LUAD with the EML4‐ALK translocation. H&E staining of patient #2 showed that the normal lung organoids had uniform basal nuclei. Tumor organoids exhibited an increased nuclear‐to‐cytoplasmic ratio, nuclear hyperchromasia, and pleomorphism. IHC staining of the tumor and matched normal lung organoids showed ALK staining in tumor organoids and the corresponding primary tissue (Figure 2B). Tumor cancer organoid #3 was LUAD with a KRAS mutation (G12C). All of the tumor organoids presented elevated galectin‐9 expression compared to that of their normal lung counterparts (Figure 2C). Gal9‐KO in tumor organoids #2 and #3, generated using the CRISPR/Cas9 system, significantly reduced the ALDH^+^/CD44^+^ TIC ratio (Figure 2D–F). An in vitro limiting dilution assay for self‐renewability showed that, compared with the lung organoid #1 and #2 controls, the Gal9‐KO group had significantly reduced sphere formation and TIC frequency (Figure 2G,H). Cell viability assays using CellTiter‐Glo reagents showed that Gal9‐KO significantly sensitized tumor organoids harboring *ALK* translocation to crizotinib (organoid #2) or those with *KRAS* mutations (organoid #3) to cisplatin (Figure 2I–J). Together, studies of cell lines and tumor organoids have indicated that galectin‐9 plays a supportive role in mediating TIC phenotypes in LUAD.

### 2.3 Secretory Galectin‐9 from Tumor Organoids Suppressed T‐cell Proliferation

**Figure 3:**
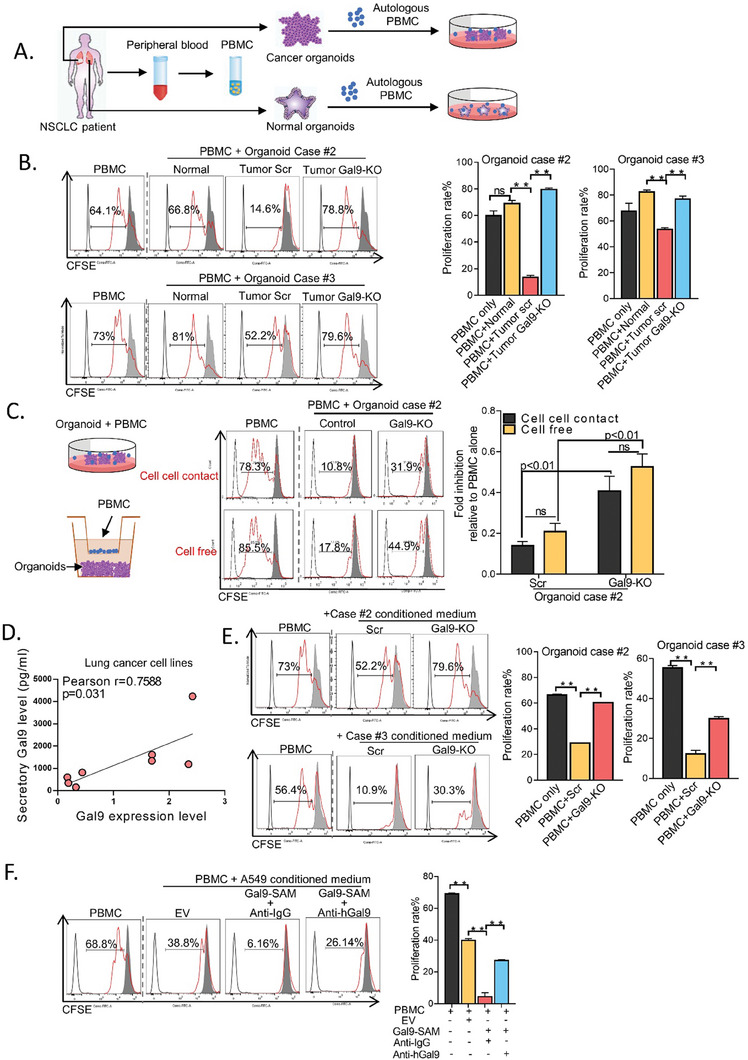
Secretary galectin‐9 from tumor organoids suppressed T cell proliferation A) Peripheral blood and lung cancer and normal lung tissues were collected from LUAD patients. PBMCs were isolated from peripheral blood and cryopreserved until the corresponding organoids were established. Coculture experiments of autologous PBMCs with normal lung organoids or LUAD organoids were performed. B) PBMCs stimulated with CD3/CD28 and stained with CFSE were cocultured with tumor or normal lung organoids from Case #2 and Case #3 at a ratio of 10:1 for 72 h. The proportions of proliferating T cells under experimental or control conditions were assessed using flow cytometry. Gray curve, unstained cells; gray area, CSFC‐stained PBMCs without CD3/CD28 stimulation; red curve, CFSE‐stained PBMCs stimulated with CD3/CD28. C) PBMCs were stained with CFSE, stimulated with CD3/CD28, and cocultured with or without organoid case #2 in the presence or absence of a transwell insert. T‐cell proliferation was detected by flow cytometry, and the red curve indicates the proportion of proliferating cells. D) Correlation between protein expression levels of galectin‐9 and the secreted levels of galectin‐9 in a panel of lung cancer cell lines. E) Effect of conditioned medium from Case#2 and Case#3 with or without galectin‐9 manipulation on T cell proliferation. F) Effect of conditioned medium from A549 cells with or without galectin‐9 overexpression or treatment with an anti‐galectin‐9 antibody on T cell proliferation. ^**^ *p* < 0.01, compared with the control by one‐way ANOVA. The data are presented as the means±SDs of three replicates. ns, not significant.

Although galectin‐9 expression in immune cells is known to mediate immune cell function, whether its expression in LUAD cells modulates the immune microenvironment has not been reported. To investigate, we first evaluated T cell proliferation using cocultures of CFSE‐stained, CD3/CD28‐stimulated autologous PBMCs and tumor or normal lung organoids, respectively (**Figure** **3**A). Flow cytometry analyses showed cocultures with tumor organoids significantly suppressed T lymphocyte proliferation compared to controls or the corresponding normal lung organoids, respectively. Knockout of galectin‐9 in tumor organoids reversed the inhibitory effects and enabled T‐cell proliferation (Figure 3B). Next, we studied whether the inhibition of T‐cell proliferation was mediated through cell‐cell contact or cell‐free mechanisms. Cancer organoid #2 was first seeded in the lower chamber, followed by autologous PBMCs being seeded into the insert or directly into the wells, creating a contact‐free or contact‐enabled environment, respectively. Under both conditions, T lymphocyte proliferation was reduced, and the levels of suppression were similar, indicating that this action occurred mainly through a cell‐free mechanism. In both assays, T cell proliferation was restored by galectin‐9 knockout (Figure 3C). These results indicated that galectin‐9 might suppress T cell proliferation as secretion rather than surface expression. To confirm the level of galectin‐9 secretion in lung cancer cells, a conditioned medium from a panel of LUAD cell lines and the normal bronchial epithelium cell line BEAS‐2B was collected. The secretory levels of galectin‐9 were tested using ELISA. The results showed that the levels of secreted galectin‐9 from lung cancer cell lines were higher than those from normal lung epithelium (Figure S2A, Supporting Information). Furthermore, Pearson correlation analysis showed that the secretory levels of galectin‐9 were positively correlated with their respective protein expression levels, as demonstrated by western blot analysis (Figure 3D; Figure S2B, Supporting Information). Functionally, the conditioned medium from organoids #2 and #3 inhibited the proliferation of PBMCs from healthy donors, but galectin‐9 knockout reversed this effect (Figure 3E). Similar results were observed in cancer cell lines, where conditioned medium from H1975 cells significantly suppressed T cell proliferation, which was restored by Gal9‐KO cells from 12.4% to 80.4%. On the other hand, overexpression of galectin‐9 in Gal9‐KO cells (Gal9‐KO/OE) rescued this inhibitory effect and suppressed the proportion of proliferating T cells to 47.5% (Figure S2C, Supporting Information). Conversely, overexpression of galectin‐9 in A549 cells accentuated the inhibition of T‐cell proliferation (Figure S2D, Supporting Information). To confirm that T‐cell inhibition was mediated through secreted galectin‐9, a neutralizing anti‐galectin‐9 antibody was added to the conditioned medium of galectin‐9‐OE A549 cells, which significantly reversed the inhibition and resumption of T‐cell proliferation (Figure 3F). Furthermore, the addition of recombinant galectin‐9 to PBMCs suppressed T‐cell proliferation, but this effect was prevented by the addition of a galectin‐9 neutralizing antibody (Figure S2E, Supporting Information). Overall, the results indicated that secretory galectin‐9 from LUAD cells was able to suppress T‐cell proliferation.

### 2.4 Tumor‐Secreted Galectin‐9 Suppressed T‐Cell Cytotoxicity by Increasing the Treg Proportion and Inducing Apoptosis in CD8^+^ T Cells In Vitro

**Figure 4:**
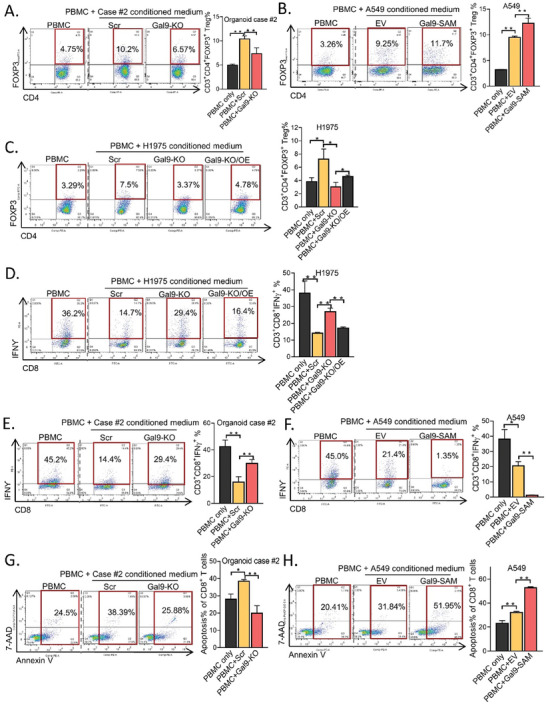
Tumor‐secreted galectin‐9 suppressed T‐cell cytotoxicity by increasing the Treg proportion and inducing apoptosis in CD8^+^ T cells. A–C) Effects of conditioned medium collected from organoid case #2 (A), A549 cells (B), and H1975 cells (C) with or without galectin‐9 manipulation on the CD3^+^/CD4^+^/FOXP3^+^ fraction of PBMCs analyzed by flow cytometry. D–F) Effects of conditioned medium collected from H1975 (D), organoid case #2 (E), and A549 (F) cells with or without galectin‐9 manipulation on the CD3^+^/CD8^+^/ IFNγ^+^ fractions of PBMCs analyzed by flow cytometry. G,H) Apoptosis of CD8^+^ T cells treated with conditioned medium from organoid case#2 (G) and A549 cells (H) with or without galectin‐9 manipulation assessed by flow cytometry using annexin V and 7‐AAD staining. ^*^ *p *< 0.05, ^**^ *p* < 0.01, compared with the control by one‐way ANOVA. The data are presented as the means±SDs of three replicates.

Regulatory T cells (Tregs) play crucial roles in suppressing T cell function. We investigated whether LUAD‐derived galectin‐9 inhibited T cell proliferation through the induction of regulatory T cells (Tregs) by flow cytometry using the markers CD3^+^/CD4^+^/FOXP3^+^. The conditioned medium from cancer organoid #2 significantly increased the CD3^+^/CD4^+^/FOXP3^+^ Treg subset compared to that of the control, while knocking out galectin‐9 suppressed this subset (**Figure** **4**A). For cancer cell lines, conditioned medium from cultured A549 cells induced an increase in the Treg proportion compared to that in PBMCs alone, and overexpression of galectin‐9 further increased the Treg proportion (Figure 4B). Treatment with conditioned medium from H1975 cells also significantly increased the Treg subset compared to that in PBMCs alone, which was reduced by Gal9‐KO but reversed by galectin‐9 overexpression (Figure 4C). Overall, the results indicated that secretory galactin‐9 from cancer organoids and cell lines might inhibit T‐cell proliferation by increasing the Treg subset. Next, the effect of galectin‐9 on the activity of IFNγ‐expressing cytotoxic T cells was investigated. Conditioned media from tumor organoids #2 and H1975 cells significantly suppressed CD3^+^CD8^+^IFNγ^+^ T cells. Knocking out galectin‐9 restored this T cell fraction, while re‐expression reversed this effect. Similarly, the A549‐conditioned medium significantly suppressed the CD3^+^CD8^+^IFNγ^+^ population, which was further attenuated by overexpressing galectin‐9 (Figure 4D–F). Hence, the results suggested that galectin‐9 secreted from LUAD cells suppressed the cytotoxic function of CD8^+^ T cells with regard to IFNγ production. We next studied whether galectin‐9 suppresses T cell cytotoxicity by inducing apoptosis in CD8^+^ T cells. Flow cytometry analysis revealed that conditioned media from cancer organoids and A549 cells significantly induced the apoptosis of CD3^+^CD8^+^ cytotoxic T cells. Galectin‐9 overexpression in A549 cells further accentuated apoptosis in CD3^+^CD8^+^ cells, but Galectin‐9 knockout in cancer organoids had the opposite effect (Figure 4G,H), indicating that secreted galectin‐9 from tumors promoted the apoptosis of cytotoxic T cells.

**Figure 5:**
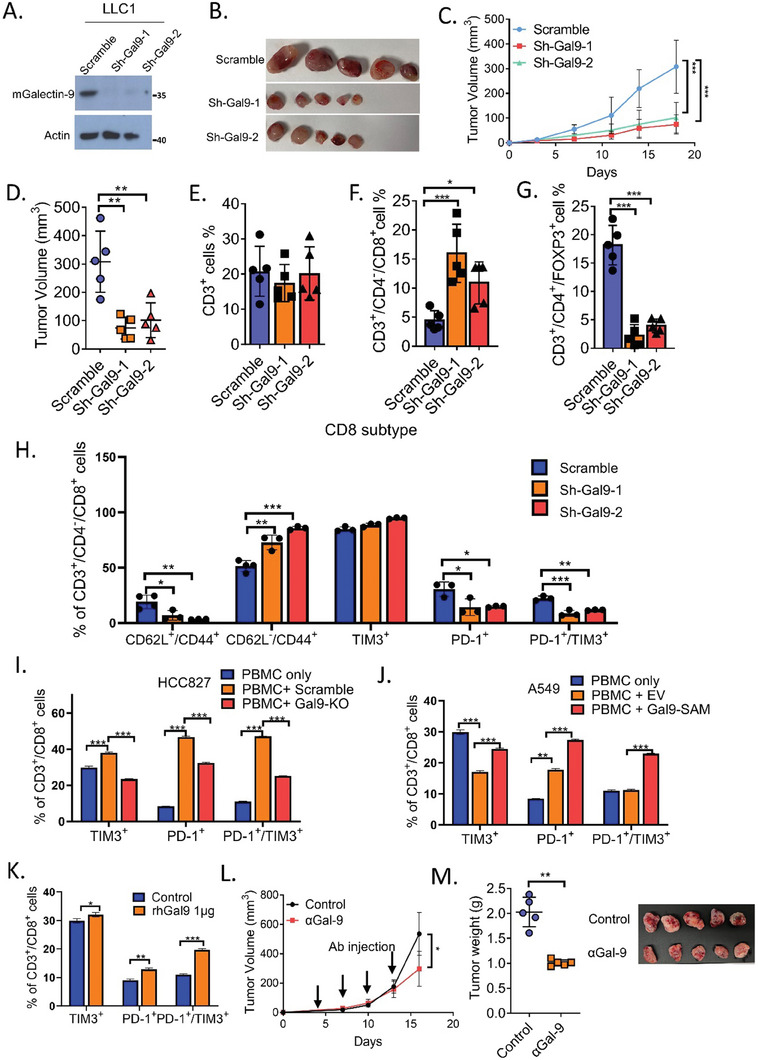
Tumor galectin‐9 increased the Treg proportion and suppressed cytotoxic T cells in vivo. A) Expression of galectin‐9 in LLC1 cells with or without Lgals9 knockdown. B–D) A total of 5 × 10^5^ LLC1 cells were subcutaneously inoculated into the flanks of C57BL mice, and the tumor volumes were monitored; *n* = 5 per group. Representative tumor images (B), tumor growth curves (C), and tumor volumes (D) are shown. ^***^ *p* < 0.001, compared with the respective control by two‐way ANOVA and corrected by Tukey's test. The error bars indicate the means ± SDs of the tumor volumes. E–G) At the endpoint of the experiment, xenografts were harvested and digested with collagenase to obtain single‐cell suspensions. Flow cytometry analysis of the infiltration of CD3^+^ T cells, CD3^+^/CD8^+^ T cells, and CD3^+^/CD4^+^/FOXP3^+^ Treg cells was performed. Proportions of infiltrated CD3^+^ T cells (E), CD3^+^/CD4^−^/CD8^+^ T cells (F), and CD3^+^/CD4^+^/FOXP3^+^ Treg cells (G) in xenografts with or without Lgals9 knockdown are shown. H) The fractions of CD8+ T cell subtypes of single‐cell suspensions derived from LLC1 xenografts with or without Lgals9 knockdown were analyzed by flow cytometry. Proportions of infiltrated CD3^+^/CD4^−^/CD8^+^/CD62L^+^/CD44^+^ T cells, CD3^+^/CD4^−^/CD8^+^/CD62L^−^/CD44^+^, CD3^+^/CD4^−^/CD8^+^/TIM3^+^, CD3^+^/CD4^−^/CD8^+^/PD‐1^+^, and CD3^+^/CD4^−^/CD8^+^/PD‐1^+^ /TIM3^+^ in xenografts are shown. I,J) Effects of conditioned medium collected from HCC827 (I) and A549 cells (J) with or without galectin‐9 manipulation on the CD3^+^/CD8^+^/TIM3^+^, CD3^+^/CD8^+^/PD‐1^+^, and CD3^+^/CD8^+^/PD‐1^+^ /TIM3^+^ fractions of CD3/CD28 stimulated PBMCs analyzed by flow cytometry. K) Effects of recombinant galectin‐9 (rhGal9) treatment on the CD3^+^/CD8^+^/TIM3^+^, CD3^+^/CD8^+^/PD‐1^+^, and CD3^+^/CD8^+^/PD‐1^+^ /TIM3^+^ fractions of CD3/CD28 stimulated PBMCs analyzed by flow cytometry. ^*^ *p *< 0.05, ^**^ *p *< 0.01, ^***^ *p *< 0.001, compared with the control analyzed by one‐way ANOVA and corrected by Tukey's test. The data are presented as the mean±SD. L,M) Effects of anti‐galectin‐9 antibody on tumor growth of LLC1 xenograft model. 5 × 10^5^ of LLC1 cells were subcutaneously inoculated into the flanks of C57BL mice. Mice bearing subcutaneous xenografts were randomly separated into two groups and treated with anti‐galectin‐9 antibody or IgG as control (150 µg/mouse, twice/week by intraperitoneal injection). Graph of tumor growth curve (L), tumor weight, and tumor image (M) were shown. ^*^ *p* < 0.05: by two‐way ANOVA. ^**^ *p* < 0. 01: by t test. The error bar indicates the mean ± SD of tumor volumes or tumor weight of five mice.

To further address the in vivo role of tumor galectin‐9 in immune modulation, a syngeneic xenograft model was constructed by injecting the mouse lung cancer cell line LLC1 with or without *Lgals9* knockdown into C57BL mice and monitoring tumor development as well as intratumoral T‐cell populations. Lgals9‐KD significantly suppressed tumor size and growth (**Figure** **5**A–D). While no significant change was observed in the total intratumoral CD3^+^ T‐cell content, Lgals9‐KD significantly suppressed the infiltration of CD3^+^/CD4^+^/FOXP3^+^ Tregs but increased the number of CD3^+^/CD8^+^ cytotoxic T cells (Figure 5E–G; Figure S3A, Supporting Information).

### 2.5 Tumor Galectin‐9 Increased the Treg Proportion and Induced Exhausted CD8^+^ T Cell Phenotypes In Vivo

Using the same syngeneic model, we further investigate whether galectin‐9 affects the differentiation of CD8^+^ T cell subtypes. The results showed that Lgals9‐KD significantly reduced the proportion of CD8^+^/CD62L^+^/CD44^+^ memory T cells but increased the proportion of CD8^+^/CD62L^−^/CD44^+^ effector T cells. PD‐1 and TIM‐3 are inhibitory receptors that regulate T‐cell exhaustion. The co‐expression of PD‐1 and TIM‐3 on T cells has been reported to define the most severely exhausted CD8^+^ T cell subsets, which dominate the tumor‐infiltrating CD8^+^ T cell population. ^[^ ^20^, ^21^ ^]^ The effect of tumor galectin‐9 on T‐cell exhaustion was also studied. It was found that Lgals9‐KD significantly reduced the proportion of CD8^+^/PD‐1^+^ T cells and that of CD8^+^/PD‐1^+^/TIM‐3^+^ T‐cells (Figure 5H; Figure S4A–G, Supporting Information). The role of tumor‐secreted galectin‐9 in CD8^+^ T cell exhaustion was further validated using PBMC activated by CD3/CD28 and conditioned medium collected from galectin‐9‐manipulated cell lines. PBMC treated with conditioned medium from HCC827 cells resulted in a significant increase in the proportion of CD8^+^/PD‐1^+^ T cell subset, CD8^+^/TIM‐3^+^ subset, and CD8^+^/TIM‐3^+^/PD‐1^+^ subset, which was reverted by knocking‐out of galectin‐9 in HCC827 (Figure 5I). In contrast, treatment with conditioned medium from A549 cells with galectin‐9 overexpression significantly induced CD8^+^/PD‐1^+^ and CD8^+^/TIM‐3^+^/PD‐1^+^ T cells compared to PBMC alone or PBMC treated with conditioned medium from control A549 cells (Figure 5J). Similar alterations were observed in PBMC treated with recombinant galectin‐9 (Figure 5K). Together, these results indicate that secretory galectin‐9 suppresses the CD8^+^ effector T cell subsets and induces the exhausted CD8^+^ T cell subset.

The therapeutic potential of targeting galectin‐9 using galectin‐9 neutralizing antibody was further investigated. Treatment with anti‐galectin‐9 antibody significantly inhibited the tumor growth and tumor weight of LLC1 xenografts (Figure 5L,M), indicating that galectin‐9 might be a druggable target for lung cancer.

Overall, the results from the in vitro and in vivo models indicated that tumor‐secreted galectin‐9 mediated immune suppressive effects on T cells by suppressing T‐cell proliferation, inhibiting the cytotoxicity of CD8^+^ T cells, promoting the apoptosis and exhaustion of CD8^+^ T cells and inducing the Treg fraction.

### 2.6 High Expression of Galectin‐9 in Human LUAD Correlated with Poor Survival

**Figure 6:**
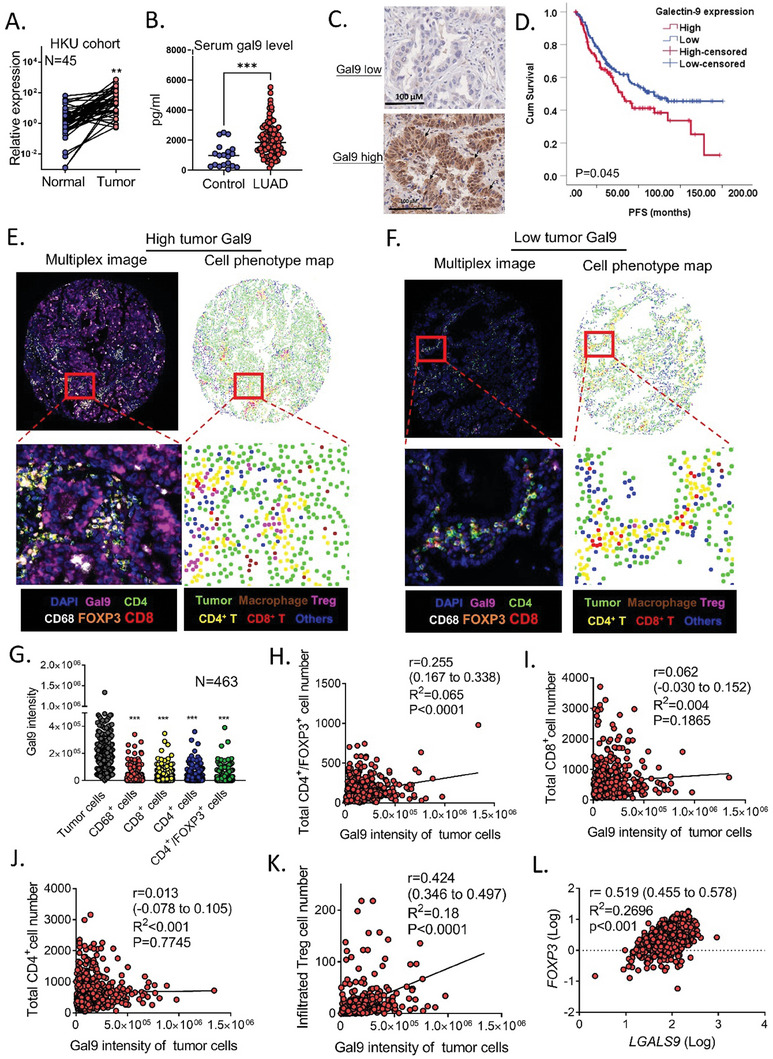
Galectin‐9 expression in tumor cells was positively correlated with Treg infiltration. A) mRNA level of *LGALS9* in paired normal/tumor lung cancer tissues from the HKU cohort. ^**^ *p* < 0.01 by paired t‐test. B) Serum galectin‐9 level from 93 LUAD patients and 19 healthy donors. ^***^ *p* < 0.001 by un‐paired t‐test. C) Representative immunohistochemistry image of galectin‐9 showing low‐grade (left) and high‐grade (right) expression in lung adenocarcinoma. D) Kaplan–Meier survival curves according to log‐rank tests of 264 resected primary LUAD samples stratified by galectin‐9 expression levels for progression‐free survival (PFS). E) Representative multiplex image (left) and the corresponding cell phenotype map of the LUAD core with high tumor galectin‐9 expression. F) Representative multiplex image (left) and the corresponding cell phenotype map of the LUAD core with low tumor galectin‐9 expression. G) Galectin‐9 intensity in different types of cells in LUAD tissue. ^***^ *p *< 0.001 compared to tumor cells analyzed by one‐way ANOVA and corrected by Tukey's test. H) Correlation between tumor galectin‐9 intensity and the total number of Treg cells analyzed by the Pearson correlation test. I) Correlations between tumor galectin‐9 intensity and the total number of CD8^+^ T cells were analyzed via Pearson correlation analysis. J) Correlation between tumor galectin‐9 intensity and the number of total CD4^+^ T cells analyzed by Pearson correlation analysis. K) Correlation between tumor galectin‐9 intensity and the number of tumor‐infiltrated Treg cells analyzed by the Pearson correlation test. L) Correlation of LGALS9 and NFATc2 mRNA levels determined by qPCR in the TCGA lung cancer dataset analyzed by Pearson correlation analysis.

To investigate the role of galectin‐9 in human lung cancer, its expression and correlation with patient survival were studied. Data from the TCGA cohort showed that *LGALS9* mRNA was significantly upregulated in LUAD compared to that in the corresponding normal lung (Figure S5A, Supporting Information), while the log‐rank test showed that high *LGALS9* mRNA levels were significantly correlated with poor overall survival (*p* = <0.001) and disease‐free survival (*p* = <0.001) (Figure S5B,C, Supporting Information). The data from a more limited local patient cohort also showed significant upregulation of *LGALS9* in LUAD compared to that in paired normal lung tissues (**Figure** **6**A, Supporting Information), with a borderline significant association with overall survival (*p* = 0.054) (Figure S5D, Supporting Information). Levels of galectin‐9 in serum from LUAD patients or healthy donors were compared using ELISA. It was shown that serum galectin‐9 levels of lung cancer patients were significantly higher than those of healthy donors (Figure 6B). To specifically evaluate tumor‐derived galectin‐9, we performed immunohistochemistry (IHC) on our cohort of excised primary LUAD samples; the results showed high galectin‐9 expression in 40.2% (106/264) of the patients (Figure 6C). Expression levels of galectin‐9 in tumors were positively and significantly correlated with the corresponding serum galectin‐9 level (Figure S5E, Supporting Information). Furthermore, high expression was significantly associated with shorter progression‐free survival (RFS) (p = 0.045) (Figure 6D). The correlation of patient serum galectin‐9 level with their prognosis was also analyzed by log‐rank test. However, results showed that serum galectin‐9 level did not correlate with survival outcome (Figure S5F, Supporting Information), indicating that tumor galectin‐9 might be a more specific prognostic marker. Overall, the results of both the TCGA and local datasets indicate that galectin‐9 is upregulated in LUAD and that a high level of tumor galectin‐9 expression predicted poor survival.

### 2.7 Tumor Expression of Galectin‐9 Correlated with Treg Infiltration in Resected Human LUAD

To further delineate the relationship between tumor cell expression of galectin‐9 and immune cell populations in clinical LUAD, multiplex fluorescent IHC was performed on tissue microarrays encompassing 463 tumor cores. Cores considered unsuitable for analysis were excluded, e.g., those showing extensive necrosis or large sheets of alveolar macrophages. Tissue and cell segmentation, cell phenotyping, cell enumeration, and galectin‐9 intensity were analyzed using inForm software trained for the identification of tumor areas (sheets or clusters of coherent cells with large pleomorphic nuclei), CD8 T cells (CD8^+^), Tregs (CD4^+^/FOXP3^+^), macrophages (CD68) and stromal cells (DAPI^+^/marker^−^) (Figure S6A, Supporting Information). Cores with high galectin‐9 expression exhibited increased infiltration of CD4^+^FOXP3^+^ Treg cells and decreased infiltration of CD8^+^ T cells, while cores with low galectin‐9 expression exhibited decreased Treg cell infiltration (Figure 6E,F; Figure S6B,C, Supporting Information). Analysis of all designated cell types revealed differential expression levels of galectin‐9, with the highest expression observed in tumor cells implicating tumor cells as the major source of galectin‐9 in LUAD (Figure 6G). When each tumor core was included in the entire analysis, a significant positive correlation (r = 0.255; R^2 ^= 0.065) was observed between the total intensity of tumor cell galectin‐9 expression and the total number of Tregs (CD4^+^/FOXP3^+^), but no significant correlations were observed for other immune cell types, including CD4^+^ T cells and CD8^+^ T cells (Figure 6H–J). Notably, when the stromal areas of each core were excluded from the analysis, an even stronger correlation was detected between tumor Gal‐9 expression and the number of tumor‐infiltrating Tregs (r = 0.424; R^2 ^= 0.18) (Figure 6K). Consistently, comparisons of *LGALS9* and *FOXP3* mRNA levels, which represent tumor galectin‐9 and Tregs, respectively, in the TCGA LUAD dataset also revealed a positive correlation (Figure 6L). Thus, at both the transcript and protein levels in different datasets, the findings suggested that tumor‐derived galectin‐9 mediated Treg infiltration in clinical LUAD.

### 2.8 *LGALS9* is an NFATc2 Target that Mediates Immune Suppression and TIC Characteristics

**Figure 7:**
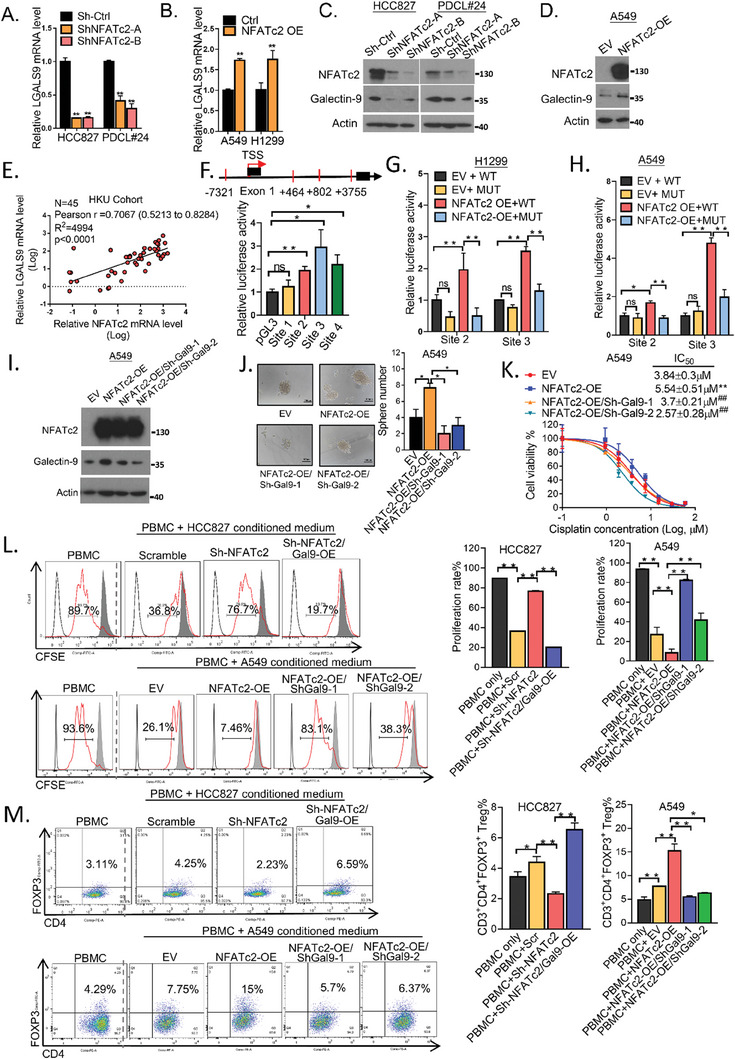
NFATc2 supported TIC phenotypes and mediated immune suppression through galectin‐9. A,B) LGALS9 mRNA expression in NFATc2‐knockdown (A) or NFATc2‐overexpressing (B) lung cancer cells determined via qPCR. C,D). (C) Protein levels of galectin‐9 and NFATc2 in NFATc2‐knockdown (C) or NFATc2‐overexpressing lung cancer cells determined by western blot (D). E) Correlation of LGALS9 and NFATc2 mRNA expression levels determined by qPCR in a local human LUAD cohort analyzed by Pearson correlation analysis. F) Computational prediction of NFAT binding sites (marked in red) in the LGALS9 regulatory region. TSS: transcription start site (upper). Luciferase activity of the LGALS9 reporters in 293T cells was determined by dual‐luciferase reporter assay. G,H) Luciferase activity of LGALS9 reporters with NFATc2‐WT or NFATc2‐MUT binding sites in H1299 cells (G) and A549 cells (H) with or without NFATc2 overexpression determined by dual luciferase reporter assay. I) Expression of galectin‐9 and NFATc2 in A549 cells with or without NFATc2 and galectin‐9 manipulation determined via western blotting. J) Sphere formation assay was performed in A549 cells with or without NFATc2/galectin‐9 manipulation. K) Cell viability assay of cisplatin performed on A549 cells with NFATc2/galectin‐9 manipulation. L) Effect of conditioned medium from HCC827 and A549 cells with or without NFATc2/galectin‐9 manipulation on T cell proliferation, as determined by flow cytometry. (M) Effect of conditioned medium from HCC827 cells and A549 cells with or without NFATc2/galectin‐9 manipulation on the CD3^+^/CD4^+^/FOXP3^+^ Treg cell proportion, as determined by flow cytometry. ^*^ *p* < 0.05; ^**^ *p* < 0.01 compared with the control. ^##^ *p* < 0.01 compared with cells with only NFATc2 manipulation by one‐way ANOVA corrected by Tukey test. The data are presented as the means±SDs of three replicates.

NFATc2 is a crucial transcription factor that regulates the expression of immune molecules, such as *FOXP3* and *IL2*. However, we have also found that NFATc2 is expressed in TIC of LUAD, and that it plays a support role.^[^ ^22^ ^]^ However, whether NFATc2 also has an immunomodulatory role when expressed in cancer cells is unknown. To investigate, we hypothesized that NFATc2 might regulate the expression of galectin‐9. We observed, via qPCR and western blot, that NFATc2 knockdown (NFATc2‐KD) decreased the expression of *LGALS9* and galectin‐9 in HCC827 and PDCL#24 cells, while NFATc2 overexpression (NFATc2‐OE) had the opposite effects on A549 and H1299 cells (**Figure** **7**A–D). In 45 patients in our clinical LUAD cohort analyzed by qPCR, the *LGALS9* and *NFATc2* mRNA levels were significantly correlated (Figure 7E). Similarly, the 539 LUAD samples from the TCGA dataset analyzed by RNA‐seq also showed a positive correlation (Figure S7A, Supporting Information), suggesting that NFATc2 could transactivate *LGALS9*. Hence, we performed in silico binding site prediction, which found 4 putative NFAT binding sites in the regulatory region of *LGALS9*: −7321 (site 1), +464 (site 2), +802 (site 3), and +3755 (site 4). The sequences of these 4 sites were subsequently cloned and inserted into the pGL3 luciferase reporter vector for luciferase reporter assays, which revealed a significant increase in the transcriptional activity of the reporters at sites 2, 3, and 4 compared to that of the vector control (Figure 7F). Furthermore, the reporter activities of *LGALS9* at sites 2 and 3 were enhanced in 293T cells engineered to overexpress NFATc2 but were significantly suppressed after mutation of these NFAT binding sites (Figure S7B, Supporting Information). Additional verification of these findings was obtained by observing increased transcriptional activity in H1299 and A549 cells stably overexpressing NFATc2 (Figure 6G,H).

Next, to identify whether galectin‐9 is a functional mediator of NFATc2‐mediated TIC enhancement and immune modulation, galectin‐9 was overexpressed in NFATc2‐knockdown cells (Sh‐NFATc2/Gal9‐OE) or knocked down in cells overexpressing NFATc2 (NFATc2‐OE/Sh‐Gal9) (Figure 7I; Figure S7C, Supporting Information). In cells transfected with Sh‐NFATc2/Gal9‐OE, tumor sphere formation, and cancer drug resistance were restored compared to those in cells with only NFATc2 knockdown (Figure S7D–F, Supporting Information). In contrast, in NFATc2‐OE/Sh‐Gal9 cells, the promotional effect of NFATc2 overexpression on the TIC density was significantly suppressed (Figure 7J‐K).

To determine whether tumor NFATc2 expression affects the immune microenvironment through galectin‐9, we investigated the effects of NFATc2 and galectin‐9 manipulation on T‐cell proliferation. First, we observed that the significant suppression of T‐cell proliferation by conditioned medium from HCC827 cells was reversed by NFATc2 knockdown, but overexpressing galectin‐9 restored this suppressive activity. Conversely, overexpression of NFATc2 in A549 cells suppressed T‐cell proliferation to a greater extent than the control, but knocking down galectin‐9 restored the proliferation of T cells (Figure 7L). Next, we studied whether NFATc2 mediated Treg differentiation through galectin‐9 and observed that NFATc2‐KD in HCC827 cells suppressed the Treg fraction, which could be reversed by overexpressing galectin‐9. In contrast, NFATc2‐OE in A549 cells induced the Treg subset, which could be suppressed by knocking down galectin‐9 (Figure 7M). In summary, these results suggest that galectin‐9 is involved in NFATc2‐mediated regulation of TIC and T lymphocyte immunosuppressive functions.

## 3 Discussion

High levels of cancer stem cell activity and an immunosuppressive microenvironment are both aggressive phenotypes that lead to poor prognosis in patients with LUAD. An increasing number of studies have revealed an association between TIC properties and immunosuppressive signatures,^[^ ^11^, ^12^ ^]^ and elucidating the interactions of these factors would help identify new treatment options to diminish TIC propagation and immune tolerance simultaneously.

Galectin‐9 is a known ligand of the immune checkpoint receptor TIM‐3 expressed in cells of both the innate and adaptive immune systems that mediates suppressive immune modulation.^[^ ^15^, ^23^ ^]^ Our computational prediction showed that galectin‐9 might be a transcriptional target of the immune regulator NFATc2. On the other hand, we and others have reported that NFATc2 expression in carcinomas contributes to tumor growth and TIC phenotypes.^[^ ^22^, ^24^, ^25^ ^]^ These findings led us to hypothesize that galectin‐9 could play dual roles in tumor propagation and modulation of the immune microenvironment. In this study, tumor‐supportive roles were addressed through the study of TICs, which are cell fractions recognized to display enhanced metastatic potential and resistance to cancer drug treatment among all cell populations in cancer. TICs were designated by their capacity for tumorsphere formation, as well as their highest coexpression of CD44 and ALDH. TICs selected by these surrogate markers displayed significantly higher galectin‐9 expression, suggesting that they are involved in tumor propagation. Indeed, manipulation of galectin‐9 expression yielded consistent in vitro and in vivo results on self‐renewal and cancer drug responses. Moreover, the cancer organoids raised for this study, which are believed to be improved investigatory cancer models, also yielded compatible results, including response to targeted therapy of cancer organoid #2 which harbors *ALK* translocation. Further support was provided by observations of clinical tumors from the TCGA cohort and our local dataset, in which high expression of galectin‐9 correlated with shorter survival. Consistent with our observation, it was reported that galectin‐9 levels were elevated in the plasma exosomes of NSCLC patients and predicted adverse clinical outcomes.^[^ ^26^ ^]^ The oncogenic role of galectin‐9 in carcinoma cells has been reported to be controversial. In cancers of the breast and liver, anti‐metastatic potentials are implicated^[^ ^27^, ^28^ ^]^; moreover, in the stomach, pancreas, and biliary tract, inhibition of proliferation or enhancement of tumor cell apoptosis is observed.^[^ ^29^, ^30^, ^31^ ^]^ In contrast, a recent study reported that galectin‐9 promotes breast cancer metastasis through activating the focal adhesion kinase (FAK) pathway.^[^ ^32^ ^]^ One study reported the involvement of galectin‐9 in TIC maintenance in leukemia,^[^ ^17^ ^]^ but its role in the TIC of solid tumors has not been reported.

Tregs are believed to play an important role in tumor progression by maintaining an immune‐tolerant microenvironment and regulating T‐cell cytotoxicity.^[^ ^33^ ^]^ High proportions of tumor‐infiltrating Tregs are recognized as negative prognostic factors in lung cancers,^[^ ^34^ ^]^ while decreased ratios of CD8^+^ T cells/Treg cells are correlated with poor prognoses in patients with breast and gastric cancers.^[^ ^35^, ^36^ ^]^ Thus, Treg regulatory pathways might provide novel treatment opportunities. To investigate whether galectin‐9 could mediate immune tolerance, cocultures of human autologous immune cells and cancer organoids and a syngeneic mouse model of lung cancer xenografts were studied, which showed that tumor‐secreted galectin‐9 suppressed T‐cell toxicity through inducing the Treg fraction and promoting CD8^+^ T‐cell apoptosis. Consistent with our findings, high expression levels of galectin‐9 correlated with infiltration of TIM3^+^Treg in chronic lymphocytic leukemia.^[^ ^37^ ^]^ Mechanistically, galectin‐9 has been reported to stabilize induced Tregs (iTregs) through binding to its receptor CD44.^[^ ^38^ ^]^ Yang and colleagues reported that treatment with an antagonistic galectin‐9 monoclonal antibody in a syngeneic mouse colon carcinoma model led to the expansion of tumor‐infiltrating cytotoxic CD8^+^ T cells as well as Tregs. These results indicate that secretory galectin‐9 functions to attenuate CD8^+^ T cells, which agrees with our observation, but the suppression of Tregs contradicts our finding.^[^ ^42^ ^]^ The researchers suggested that galectin‐9 mediates its suppressive effect through TIM3 expressed on Tregs, leading to their death. However, since TIM3^+^ Tregs constitute the major fraction of tumor‐infiltrating Tregs and provide the bulk of tumor immunosuppressive activity, permitting tumor progression in vivo,^[^ ^39^ ^]^ it is intriguing to suggest that galectin‐9 has a predominant suppressive rather than promotional effect on Tregs, as we have observed. Notably, according to our multiplex IHC results, carcinoma cells, rather than immune cells, displayed the strongest galectin‐9 expression. Since the immune modulatory effects of tumor cell‐derived galectin‐9 have not been previously reported, our findings might be representative of in vivo interactions in the clinical setting. These contradictory results might also imply that galectin‐9 and TIM3 play complex and variable roles in different T‐cell subsets across different cancers. Further studies are needed to clarify this controversy. Our study demonstrated that tumor secreted galectin‐9 suppressed CD8^+^ T cell cytotoxicity through suppression of proliferation and induction of the PD‐1^+^ or PD‐1^+^/TIM3^+^ subset. However, the underlying mechanism regarding how galectin‐9 induces the dysfunction of CD8^+^ T cells remains elusive. One possibility is that galectin‐9 suppresses CD8^+^ T cell function through Treg induction. Besides, it has been reported that galectin‐9 can interact with PD‐1, which helps to stabilize the PD‐1^+^/TIM3^+^ exhausted T cell subset in the tumor microenvironment.^[^ ^40^ ^]^ Therefore galectin‐9 might also induce CD8^+^ T cell exhaustion through up‐regulation of PD‐1, which is worthy for future study.

Our studies revealed the dual role of galectin‐9 in Treg induction and TIC maintenance. However, the correlation between Tregs and galectin‐9‐induced TIC phenotypes remains elusive. Regulatory effects of Tregs on TIC phenotypes have been observed in leukemia through the secretion of IL10 and activation of PI3K/AKT signaling.^[^ ^41^ ^]^ In solid tumors, Tregs have been reported to induce TIC phenotypes in breast cancer, but the underlying mechanisms remain unknown.^[^ ^42^ ^]^ Whether Tregs play a potential role in regulating the TIC plasticity of LUAD should be further investigated in future studies. In addition to T cells, galectin‐9 has been reported to interact with different immune molecules. For example, in pancreatic cancer, galectin‐9 expression on myeloid cells induces immune tolerance through binding to Dectin‐1 on macrophages.^[^ ^43^ ^]^ Besides, galectin‐9 has been reported to induce the maturation of human monocyte‐derived dendritic cells (DCs).^[^ ^44^ ^]^ A recent study in colorectal cancer reported that the expression level of galectin‐9 positively correlated with infiltration of mature DCs and increased infiltration of CD8^+^ T cells, indicating the supportive role of galectin‐9 in anti‐tumor immunity of colorectal cancer.^[^ ^45^ ^]^ However, the role of galectin‐9 in cancer immune may vary amongst different cancer types. Whether galectin‐9 could regulate DC function in lung cancer remains unclear. Our study uncovered the role of galectin‐9 in regulating T‐cell function. Since DC is a crucial mediator of T cell activity, it is worthy to investigate the regulatory role of galectin‐9 in DCs and dissect the role of galectin‐9 in DC‐mediated T cell activity in future studies. In small‐cell lung cancer, galectin‐9 expression in tumor‐infiltrating lymphocytes positively correlated with PD1, CD3, CD8, and FOXP3.^[^ ^46^ ^]^ Overall, these studies indicate that galectin‐9 plays a comprehensive role in supporting the immunosuppressive tumor microenvironment.

Recent studies have reported different prognostic values of NFATc2 in cancer, but our own investigations have shown that NFATc2 supports TIC functions in LUAD and that high NFATc2 expression predicts adverse outcomes. On the other hand, NFATc2 is well known to regulate innate and adaptive immunity through mechanisms such as transcriptional regulation of FOXP3 in Treg cells and promotion of cytotoxic T‐cell exhaustion.^[^ ^47^, ^48^ ^]^ However, its role in LUAD with regard to possible immune suppression has not been determined. In the present study, NFATc2 binding sites were identified in intron 1 of *LGALS9* (a galectin‐9 coding gene), through which transcription was regulated, which in turn affects TIC promotion, self‐renewal, and drug resistance. The functional role of the NFATc2/galectin‐9 axis in LUAD was further illustrated by its suppressive effect on T‐cell proliferation and Treg induction. Thus, this study elucidated the dual roles of NFATc2/galectin‐9 in sustaining TIC phenotypes and mediating immune suppression, suggesting that galectin‐9 is a potential prognostic marker and treatment target in LUAD.

## 4 Experimental Section

### Cell Lines

Established human LUAD cell lines (H1299, A549, H1975, HCC827, H441, and 293T) and the murine lung cancer cell line LLC1 were obtained from the ATCC. Patient‐derived cell lines (PDCL#24 and TQ21) were generated from resected lung cancer or malignant pleural effusions and only cells from the 1st to 10th passages were used for the study. All procured cell lines used in this study were authenticated using the AmpFlSTR Identifiler PCR Amplification (Thermo Fisher Scientific, Waltham, MA).

### Patient Samples

Peripheral blood, primary lung cancer tissue, and corresponding normal lung tissue were obtained from ethnic Chinese patients who provided informed written consent and underwent surgical resection at Queen Mary Hospital, Hong Kong. The peripheral blood and tissue collection protocols used were approved by the HKU/HAHKWC Institutional Review Board. Clinico‐pathological parameters of LUAD patients involved in this study are provided in Table S1 (Supporting Information).

### Human Peripheral Blood Mononuclear Cell (PBMC) Isolation

Human peripheral blood mononuclear cells (PBMCs) were prepared from buffy coats obtained from healthy donors from the Hong Kong Red Cross or from lung cancer patients using Ficoll‐Hypaque density gradient centrifugation (Sigma‒Aldrich, St. Louis, MO).

### Establishment of Organoid Culture

Fresh lung cancer or normal lung tissue was processed for cell isolation and long‐term expansion as previously described.^[^ ^49^ ^]^ Briefly, fresh solid tissues were minced and digested in lung organoid medium (LOM) (Advanced DMEM/F12 with 1% GluaMax, 1% 1 M HEPES and 1% penicillin/streptomycin) supplemented with 10% Rspol conditional medium, 10% Noggin conditional medium (a kind gift from Prof Hans Clevers), 1 X B27, 1.25 mM N‐acetylcysteine, 10 mM nicotinamide, 5 µM ROCK inhibitor, 500 nM ALK inhibitor, 1 µM p38 MAP kinase inhibitor, 5 ng mL^−1^ FGF‐7 and 20 ng mL^−1^ FGF‐10) containing 1 mg mL^−1^ collagenase (Sigma‒Aldrich) on a shaker at 37 °C for 1 h. Digestion was stopped by the addition of FBS. The digested tissue suspensions were sequentially sheared using 10 and 5 mL plastic pipettes. Afterward, the suspensions were sequentially strained through 100 µm and 70 µm filters and centrifuged at 400 × g. The visible red pellet was lysed by using 3 mL of red blood cell lysis buffer and centrifuged again at 400 × g. The pellet was subsequently resuspended in cold growth factor reduced‐basement membrane matrix (GFR‐BME) (Corning), and 40 µL of cell suspension was seeded in a prewarmed 24‐well suspension culture plate (Greiner) at 37 °C for 20 min. After gelation, 500 µL of LOM was added to each well. Lung cancer organoids were distinguished from normal cystic organoids by their pleomorphic cell morphology. Information about primary tissue specimens obtained from LUAD patients for organoid culture is provided in Table S2 (Supporting Information).

### Plasmids

shRNAs targeting human NFATc2 and LGALS9 were purchased from Sigma‒Aldrich (St. Louis, MO). Luciferase reporter vectors were purchased from GeneScript (NJ, USA). Site‐directed mutagenesis of the consensus NFAT binding site (GGAAA to GACTA) on reporters was performed using QuikChange (Stratagene). For CRISPR/Cas9 knockout of LGALS9, a sgRNA targeting LGALS9 (GACTATTCAAGGAGGTCTCC) was cloned and inserted into the inducible split Cas9 vector pLSC5 purchased from Addgene (Cambridge, MA; http://www.addgene.org) as described previously.^[^ ^50^ ^]^ For endogenous overexpression of galectin‐9, a sgRNA targeting the promoter of LGALS9 was cloned and inserted into the lenti sgRNA(MS2)_zeo vector purchased from Addgene as previously described.^[^ ^51^ ^]^

### Sphere Formation Assay

For sphere formation assays, 250 A549, 500 HCC827, and 1000 H1975 cells were seeded in low‐attachment plates (Costar) with cancer stem cell (CSC) medium (RPMI 1640 medium supplemented with 20 ng mL^−1^ EGF, 20 ng mL^−1^ FGF, 40 ng mL^−1^ IGF and 1 X B27) for 10 days.

### In Vitro Limiting Dilution Assay

Patient‐derived organoids were trypsinized into single cells. Decreasing numbers of cells (250, 100, 50, and 10) were seeded into 96‐well low‐attachment plates. The cells were cultured in a CSC medium for 14 days. The number of wells containing spheres was recorded, and the in vitro CSC frequency was calculated by the online tool http://bioinf.wehi.edu.au/software/elda/. ^[^ ^52^ ^]^

### Cell Viability Assay

For lung cancer cell lines, cells were seeded in 96‐well plates and incubated at 37 °C for 24 h, followed by incubation with 200 µL of medium containing escalating doses of the respective drugs, gefitinib (Selleckchem), crizotinib (Selleckchem) or cisplatin (Sigma‒Aldrich), for 72 h. To assess the half maximal inhibitory concentration (IC_50_), 5 mg mL^−1^ MTT (Sigma‒Aldrich) was added, and the mixture was incubated at 37 °C for 4 h. The absorbance was determined at 570 nm. For organoid assays, organoids were dissociated by mechanical shearing, strained through a 70 µm filter, and resuspended in LOM medium containing 5% GFR‐BME; finally, 30 µL of suspension was seeded into 384‐well plates. Organoids were treated with the indicated drugs at various doses for 5 days. Cell viability was determined with a CellTiter‐Glo 2.0 assay kit (Promega), and luminescence was measured with a multifunctional reader. The drug response curve was plotted, and the IC_50_ was calculated using a nonlinear regression model in GraphPad Prism 7.0.

### Flow Cytometry Analysis

For ALDH/CD44 staining, ALDH activity was detected with an ALDEFLUORTM kit (Stem Cell Technologies) according to the manufacturer's instructions. CD44 expression was detected with an anti‐CD44‐APC antibody (BD Biosciences) as previously described.^[^ ^18^ ^]^ For the analysis of cell surface proteins, the cells were incubated with antibodies in a staining wash buffer for 30 min in the dark. For intracellular proteins (FOXP3 and IFNγ), cells were fixed, permeabilized, and stained using a Transcription Factor Staining Buffer Set (eBioscience). The cells were then washed and resuspended in 0.5 mL of staining buffer. The antibodies used included APC‐anti‐human CD3, PE‐anti‐human CD4, FITC‐anti‐human CD8, PE‐anti‐human IFNγ, FITC‐anti‐human FOXP3, BV785‐anti‐human PD‐1, BV605‐anti‐human TIM3, APC/CY7‐anti‐mouse CD45, Percp5.5‐anti‐mouse CD3, FITC‐anti‐mouse CD4, PE/CY7‐anti‐mouse CD8a, and pacific blue‐anti‐mouse FOXP3, Brilliant Violet 785 anti‐mouse/human CD44, Brilliant Violet 605 anti‐mouse CD279 (PD‐1), PE/Cyanine7 anti‐mouse CD366 (Tim‐3), PE anti‐mouse CD62L, APC/Fire 750 anti‐mouse CD3, Brilliant Violet 510 anti‐mouse CD8a, Alexa Fluor 700 anti‐mouse CD45. All antibodies used were purchased from Biolegend. The cells were analyzed using a BD LSRFortessa Cell Analyzer (BD Biosciences). The results were analyzed using FlowJo software (Tree Star).

### Dual‐Luciferase Reporter Assay

Cells were transfected with expression plasmids, pRL‐TK, and reporter plasmids using Lipofectamine 2000 and cultured at 37 °C for 48 h. Luciferase activities were subsequently measured via the Dual‐Luciferase Reporter Assay System (Promega) according to the manufacturer's instructions. The luminescent signals emitted from Firefly and Renilla luciferases were recorded by a multifunctional reader.

### T Cell Proliferation Assay

PBMCs were activated with 5 µg mL^−1^ prebound anti‐CD3/anti‐CD28 antibodies (BioLegend), followed by CFSE labeling (Invitrogen) according to the manufacturer's instructions. The activated PBMCs were cocultured with organoids and treated with a conditioned medium from organoids or lung cancer cell lines. After 96 h, the cells were stained with an APC‐conjugated anti‐CD3 antibody (BioLegend), and T cell proliferation was determined by assessing CFSE intensity via flow cytometry.

### Apoptosis

Apoptosis was quantified by Annexin V‐PE and 7‐AAD staining as previously described. ^[^ ^53^ ^]^

### Tumor Microarray and Immunohistochemistry (IHC)

At least 2 tumor cores from each patient were selected and assembled into tissue microarray blocks (TMAs). IHC was performed according to routine procedures after antigen retrieval via a scientific microwave at 95 °C for 15 min in pH 8.0 EDTA. Primary antibodies against galectin‐9 (1:2000; Sigma #HPA019869) were incubated overnight at 4 °C, followed by incubation with secondary antibodies conjugated with polymer‐linked HRP (DAKO, Agilent) for 30 min at room temperature. The expression levels of galectin‐9 were scored according to the extent and intensity of cytoplasmic staining in the tumor cells. The staining intensity was graded as 1, 2, or 3 according to whether cytoplasmic staining was absent, weak, moderate, or strong, respectively. The extent of the expression was graded as 1, 2, or 3 according to whether the highest expression was observed in scattered individual cells, in aggregates of ≥5 but ≤100 cells, or in sheets of >100 cells. The products of the 2 grades were then computed, and patients with scores ≥4 were considered to have high‐level expression.

### Multiplex Fluorescence IHC Staining

Multiplex immunofluorescence staining of formalin‐fixed paraffin‐embedded tissues was performed on TMAs using an Opal Immunology Discovery Kit (Akoya biosciences) according to the manufacturer's instructions. Briefly, sections were deparaffinized using xylene and progressively hydrated with ethanol, followed by a distilled water wash. Microwave treatment was performed in AR6 buffer for antigen retrieval. Then, the slides were sequentially stained with the following primary antibodies and fluorescent dyes: anti‐CD4 (Akoya biosciences)/Opal‐520, anti‐CD8 (Akoya biosciences)/Opal‐570, anti‐FOXP3 (Biolegend, San Diego, CA, USA)/Opal‐620, anti‐CD68 (Akoya biosciences)/Opal‐650, and anti‐galectin‐9 (Sigma)/Opal‐690. For each molecule being detected, the slides were incubated with the specific primary antibody or secondary antibody, followed by Opal fluorophore solution and incubation for 10 min at room temperature. Afterward, the samples were subjected to microwave treatment again to remove the specific primary antibody. The steps were repeated for subsequent primary antibodies to achieve multiplex immunofluorescent (IF) staining. After antibody staining, the slides were incubated in DAPI solution for 5 min at room temperature, washed several times, and then mounted with coverslips. Digital images of all the TMA cores were acquired using the Vectra Polaris Imaging System and analyzed with inForm software (Akoya Biosciences Hopkinton, MA).

### Enzyme‐Linked Immunosorbent Assay (ELISA)

Serum galectin‐9 levels or secreted galectin‐9 levels from lung cancer cell lines were assessed using the Human galectin‐9 ELISA Kit (Jianglai Biological, China) according to the manufacturer's instructions.

### In Vivo Subcutaneous Xenograft Assay

All animal experiments were carried out strictly according to guidelines approved by the Animal Ethics Committee of The University of Hong Kong. The designated numbers of cells were suspended in 50 µL of cold RPMI 1640 serum‐free medium and gently mixed with the same volume of Matrigel (BD Pharmingen). The cell suspension was subsequently injected subcutaneously into the flanks of 4‐week‐old severe combined immunodeficiency (SCID) mice. For syngeneic mouse tumor models, 5 × 10^5^ LLC1 cells with or without Lgals9 knockdown were resuspended in 50 µL of cold serum‐free DMEM and 50 µL of Matrigel and were subcutaneously inoculated into 4‐ to 6‐week‐old C57BL/6J mice. Tumor volumes were measured twice weekly using calipers. Tumor volume was calculated using the formula [W^2^ × L]/2 and was expressed in mm^3^. At the end of the experiment, xenografts from the syngeneic mouse model were harvested, minced into small pieces, dissociated with collagenase, and treated with red blood cell lysis buffer. Afterward, the single‐cell suspensions were subjected to flow cytometry analysis.

For in vivo anti‐body treatment assay, 5 × 10^5^ LLC1 cells were resuspended in 50 µL of cold serum‐free DMEM and 50 µL of Matrigel and were subcutaneously inoculated into 4‐ to 6‐week‐old C57BL/6J mice. The mice were randomly separated into 2 groups, and treated with either IgG control or anti‐galectin‐9 antibody (150 µg/mouse, twice/week) (Bio X cell). All drugs were treated by intraperitoneal injection. The tumor volume and body weight were measured twice weekly. Tumor volume was calculated using the formula [W^2^ × L]/2 and was expressed in mm^3^.

### Statistics

The data were analyzed with SPSS (version 26.0; SPSS, Inc., Chicago, IL, USA), GraphPad Prism 7.0, or Excel (Microsoft, Redmond, WA, USA) software packages and were presented as the means ± standard deviations (SDs). Differential expression between paired tumor/normal tissues was analyzed by the Wilcoxon signed‐rank test. Differences between the two groups were analyzed by t‐tests for continuous variables. Differences between multiple groups were analyzed by one‐way ANOVA. Differences between the growth curves of the xenograft model were analyzed by two‐way ANOVA. Correlations between expression levels between groups were analyzed by the Pearson correlation test. Associations between galectin‐9 expression and overall survival and recurrence‐free survival were analyzed by the Kaplan–Meier method with the log‐rank test. Two‐sided *p* values <0.05 were considered statistically significant.

## Conflict of Interest

The authors disclose no potential conflicts of interest.

## Author Contributions

Z.J.X., S.Q.W., and J.J.C. contributed equally to this work. Z.J.X. and M.P.W. conceived the project idea and acquired funding support; Z.J.X., S.Q.W., J.J.C, Y.L, Y.C.J, J.L, H.Y.H and V.P.C.T. performed the experiments and analyzed the data; J.W.P.Y. and Y.H.P. supervised and contributed to the experimental design. Z.J.X., P.Y.H., and J.W.P.Y. drafted and finalized the manuscript. Z.J.X. designed and supervised the overall execution of the studies.

## Supporting information

Supporting Information

## Data Availability

The data that support the findings of this study are available from the corresponding author upon reasonable request.
