# Supplementary material for: The Dual Role of the NFATc2/galectin‐9 Axis in Modulating Tumor‐Initiating Cell Phenotypes and Immune Suppression in Lung Adenocarcinoma
Source: Adv Sci (Weinh). 2024 Mar 25;11(20):2306059. doi: 10.1002/advs.202306059 (PMC11132051; doi:10.1002/advs.202306059)
Supplement: Supplementary file 1 — Supporting Information [file ADVS-11-2306059-s001.pdf]

ADVANCED SCIENCE
Open Access

Supporting Information

for *Adv. Sci.*, DOI 10.1002/advs.202306059

The Dual Role of the NFATc2/galectin-9 Axis in Modulating Tumor-Initiating Cell Phenotypes and Immune Suppression in Lung Adenocarcinoma

Zhi-Jie Xiao, Si-Qi Wang, Jun-Jiang Chen, Yun Li, Yuchen Jiang, Vicky Pui-Chi Tin, Jia Liu, Huiyi Hu, Maria Pik Wong, Yihang Pan* and Judy Wai Ping Yam**

Table S1

Clinico-Pathological information of clinical samples

**Clinico-Pathological Variables**		**n**	**%**
**Gender**	**Female**	**178**	**67.4**
	**Male**	**86**	**32.6**
**Age (Years)**	**≦65**	**105**	**39.8**
	**＞65**	**159**	**60.2**
**Smoking History**	**Non-smoker**	**233**	**88.3**
	**Smoker**	**31**	**11.7**
**TKI[a] treatment**	**No**	**171**	**64.8**
	**Yes**	**58**	**22**
	**N/A[b]**	**35**	**13.2**
**Pathological (TNM) stage**	**I**	**156**	**59.3**
	**II**	**67**	**25.5**
	**III**	**36**	**13.7**
	**IV**	**4**	**1.5**

a: Tyrosine kinase inhibitors

b: information not available

Table S2

LUAD patient information of specimens obtained for organoid culture

	Sample name		
	Organoid case #1	Organoid case #2	Organoid case #3
Age	74	69	62
Sex	Female	Male	Male
Smoking history	Non-smoker	Non-smoker	Chronic-smoker
Histological subtype	Adenocarcinoma	Adenocarcinoma	Adenocarcinoma
Differentiation status	Poorly differentiated	Moderately differentiated	Moderately differentiated
Pathological stage	IIIA	IIA	IIB
Driver mutation	None	ALK translocation	KRAS

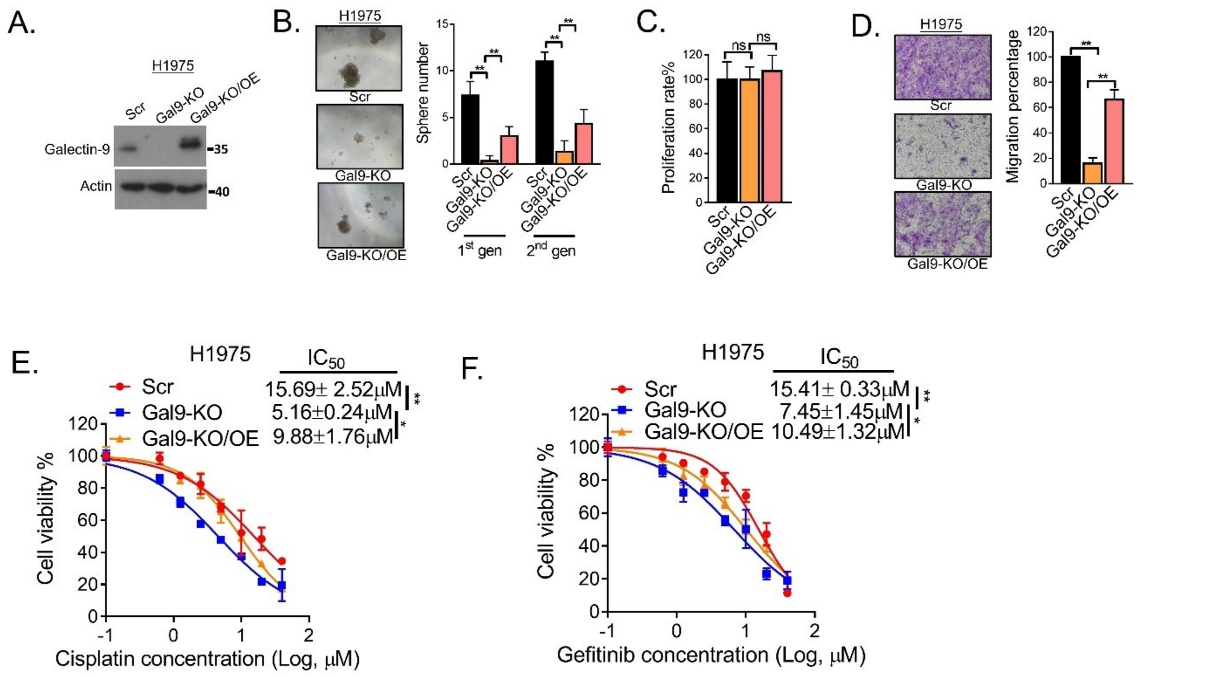

Figure S1. Galectin-9 supported the TIC phenotypes of lung adenocarcinoma. (A) Expression of galectin-9 in H1975 cells with galectin-9 knockout alone or with additional galectin overexpression, as determined by western blotting. (B) Sphere formation assay in H1975 cells with galectin-9 manipulation. (C) BrdU proliferation assay of H1975 cells with galectin-9 manipulation. (D) Migration assay of H1975 cells with galectin-9 manipulation. (E-F) Effects of galectin-9 knockout on cisplatin (E) and gefitinib (F) sensitivity in H1975 cells determined by MTT assay. *p<0.05; **p<0.01 compared to the respective control, analyzed by Student's t test for 2 group samples and one-way ANOVA for multiple group samples. The data are presented as the means±SDs of three replicates.

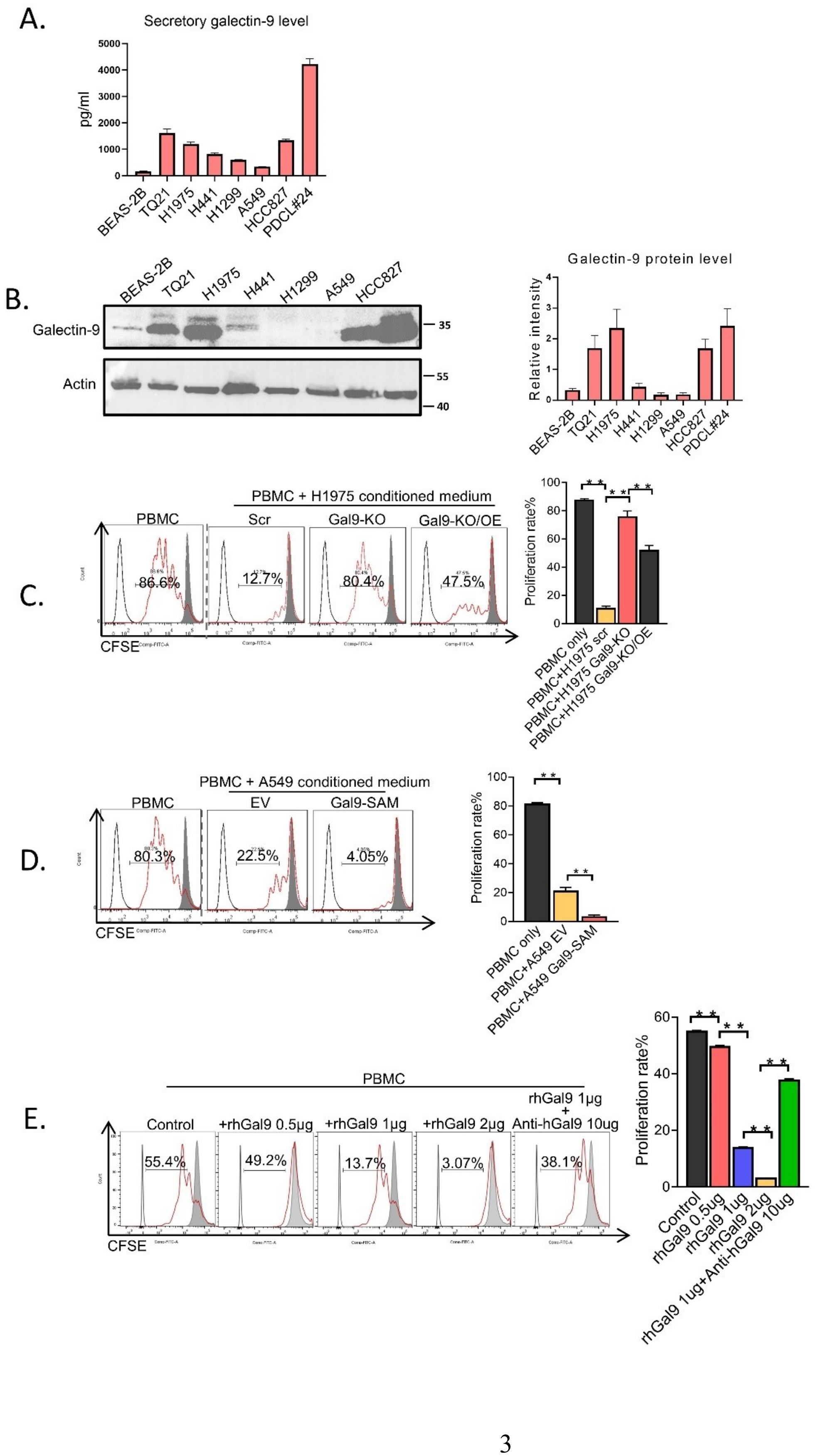
A.
Secretory galectin-9 level
pg/ml
BEAS-2B
TQ21
H1975
H441
H1299
A549
HCC827
PDCL#24
B.
Galectin-9
Actin
Galectin-9 protein level
Relative intensity
C.
PBMC + H1975 conditioned medium
PBMC
Scr
Gal9-KO
Gal9-KO/OE
86.6%
12.7%
80.4%
47.5%
CFSE
Proliferation rate%
PBMC only
PBMC+H1975 scr
PBMC+H1975 Gal9-KO
PBMC+H1975 Gal9-KO/OE
D.
PBMC + A549 conditioned medium
PBMC
EV
Gal9-SAM
80.3%
22.5%
4.05%
CFSE
Proliferation rate%
PBMC only
PBMC+A549 EV
PBMC+A549 Gal9-SAM
E.
PBMC
Control
+rhGal9 0.5µg
+rhGal9 1µg
+rhGal9 2µg
rhGal9 1µg + Anti-hGal9 10ug
55.4%
49.2%
13.7%
3.07%
38.1%
CFSE
Proliferation rate%
Control
rhGal9 0.5ug
rhGal9 1ug
rhGal9 2ug
rhGal9 1ug+Anti-hGal9 10ug

Figure S2. Tumor-secreted galectin-9 suppressed T-cell proliferation. (A) Secretory galectin-9 level of condition medium collected from lung cancer cell line panel and the normal bronchial epithelium cell line BEAS-2B as determined by human galectin-9 ELISA assay. (B) Protein level of glectin-9 from lung cancer cell line panel and the normal bronchial epithelium cell line BEAS-2B as determined by western blot. Representative image (left), and the relative intensity of galectin-9 expression normalized to actin as analyzed by ImageJ software (right). (C) Effect of conditioned medium from H1975 cells with or without galectin-9 manipulation on T cell proliferation. (D) Effect of conditioned medium from A549 cells with or without galectin-9 manipulation on T cell proliferation. (E) Effect of recombinant human galectin-9 protein and neutralizing anti-galectin-9 antibody on T cell proliferation. **$p<0.01$ compared to the respective control, analyzed by one-way ANOVA for multiple group samples. The data are presented as the means±SDs of three replicates.

A.

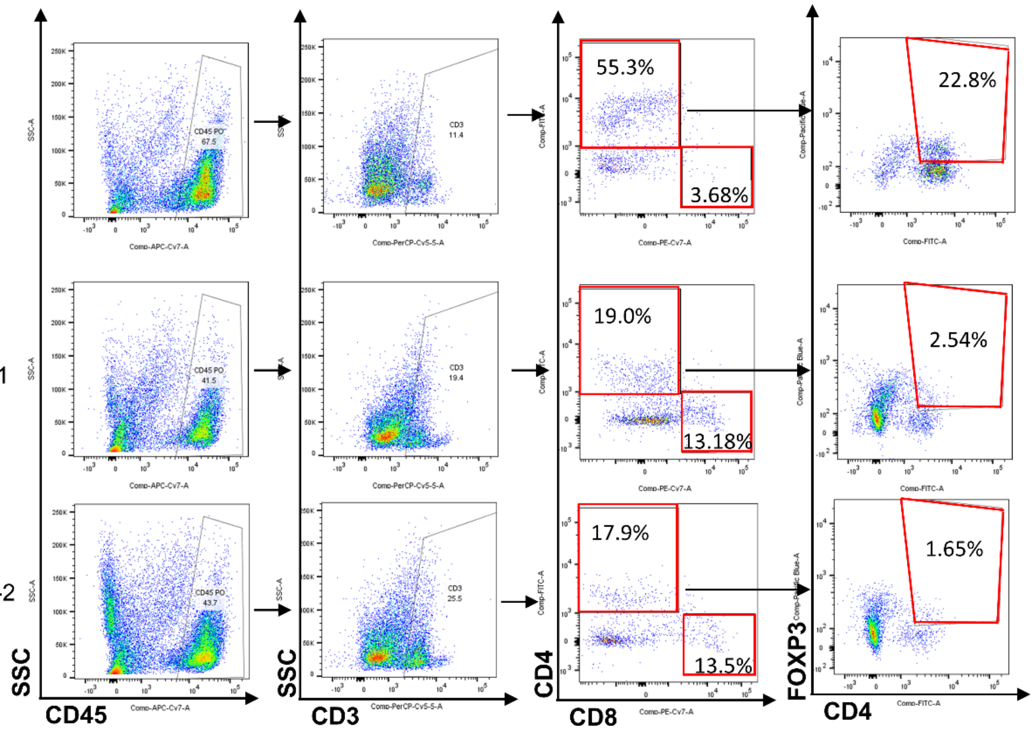

Figure S3. Tumor galectin-9 increased the Treg proportion and suppressed cytotoxic T cells *in vivo.* (A) Flow cytometry gating strategy and representative scatter plot of intratumoral T-cell subsets in syngeneic xenografts of LLC1 cells with or without Lglas9 knockdown.

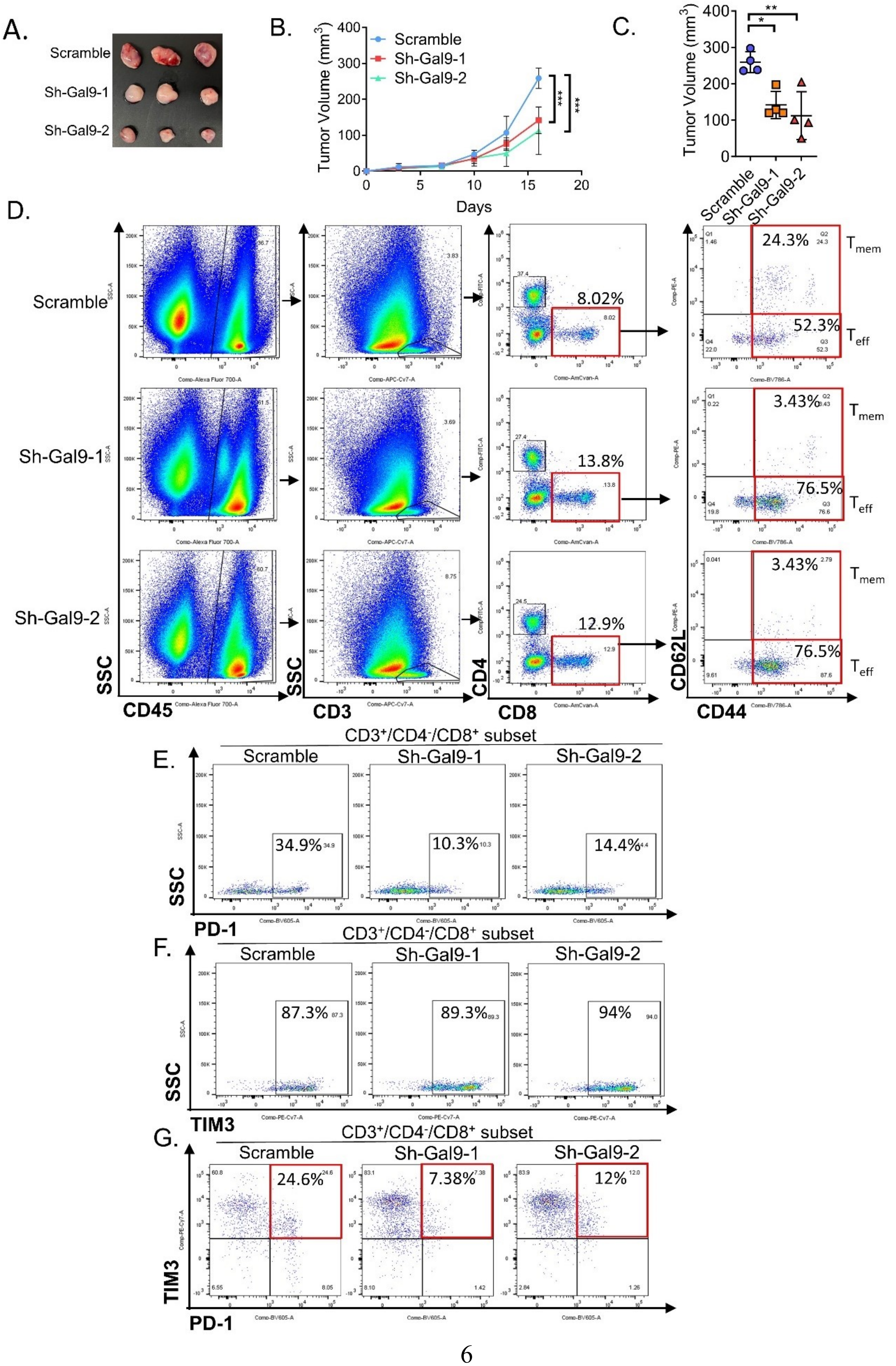
A.
Scramble
Sh-Gal9-1
Sh-Gal9-2
B.
Tumor Volume (mm3)
Days
Scramble
Sh-Gal9-1
Sh-Gal9-2
C.
Tumor Volume (mm3)
Scramble
Sh-Gal9-1
Sh-Gal9-2
D.
Scramble
Sh-Gal9-1
Sh-Gal9-2
SSC
CD45
CD3
CD4
CD8
CD62L
CD44
8.02%
13.8%
12.9%
24.3%
52.3%
3.43%
76.5%
3.43%
76.5%
Tmem
Teff
E.
CD3+/CD4-/CD8+ subset
Scramble
Sh-Gal9-1
Sh-Gal9-2
34.9%
10.3%
14.4%
SSC
PD-1
F.
CD3+/CD4-/CD8+ subset
Scramble
Sh-Gal9-1
Sh-Gal9-2
87.3%
89.3%
94%
SSC
TIM3
G.
CD3+/CD4-/CD8+ subset
Scramble
Sh-Gal9-1
Sh-Gal9-2
24.6%
7.38%
12%
TIM3
PD-1

Figure S4. Tumor galectin-9 increased suppressed cytotoxic T cells and induced $CD8^+$ T cell exhaustion. (A-C) To investigate the effect of tumor galectin-9 on tumor infiltrating $CD8^+$ T cell subsets, a syngeneic mouse model was used. A total of $5x10^5$ LLC1 cells were subcutaneously inoculated into the flanks of C57BL mice, and the tumor volumes were monitore. Representative tumor images (A), tumor growth curves (B) and tumor volumes (C) are shown. ***p<0.001, compared with the respective control by two-way ANOVA and corrected by Tukey's test. The error bars indicate the means ± SDs of the tumor volumes. (D-G) At the endpoint of the experiment, xenografts were harvested and digested with collagenase to obtain single-cell suspensions. Flow cytometry gating strategy and representative scatter plot of intratumoral infiltrated $CD3^+/CD4^-/CD8^+/CD62L^+/CD44^+$ T cells, $CD3^+/CD4^-/CD8^+/CD62L^-/CD44^+$ T cells (D), $CD3^+/CD4^-/CD8^+/PD\text{-}1^+$ T cells (E), $CD3^+/CD4^-/CD8^+/TIM3^+$ T cells (F), and $CD3^+/CD4^-/CD8^+/PD\text{-}1^+/TIM3^+$ T cells (G) in xenografts are shown.

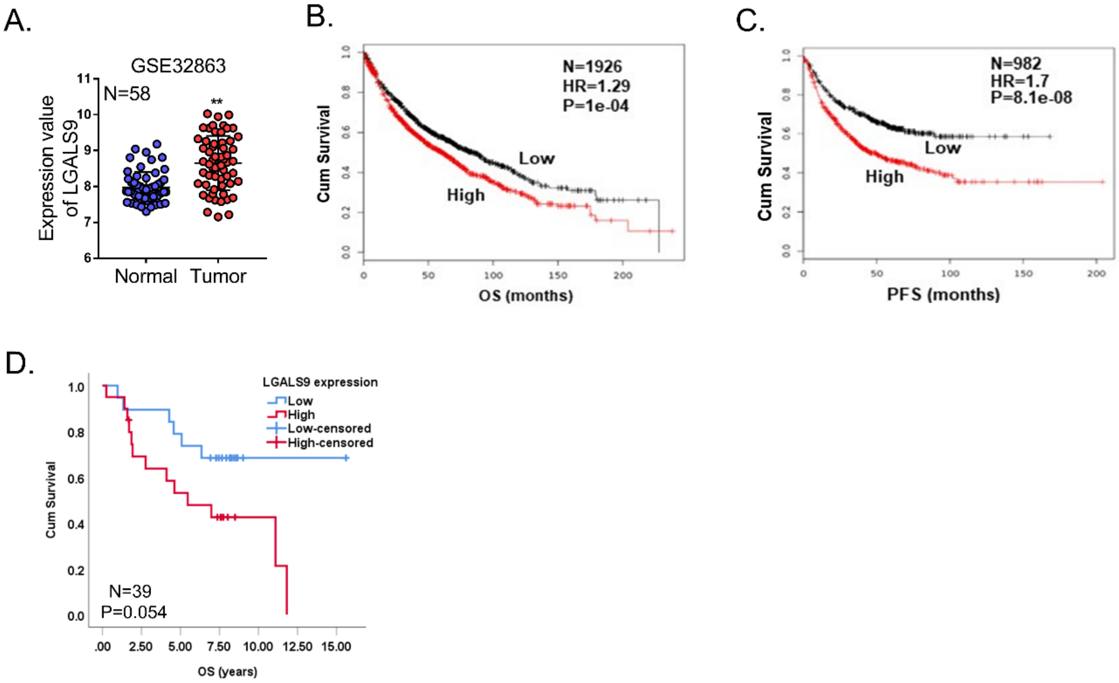

E. Correlation of serum galectin-9 level and tumor cell galectin-9 level in lung adenocarcinoma

	Tumor cell galectin-9				
	High %	Low	High	Pearson R	p value
Serum galectin-9					
Low	14.10%	31	12	0.215	0.046
High	24.7%	21	21		

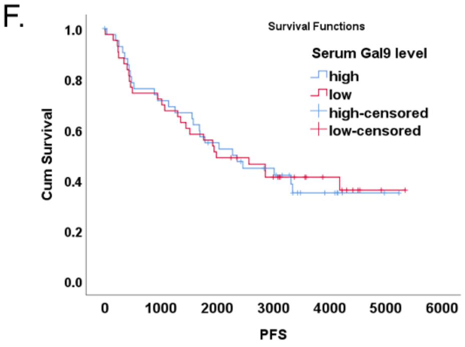

Figure S5. (A) mRNA level of LGALS9 in paired normal/tumor lung cancer tissues from lung cancer data from the TCGA dataset. (B-C) Increased LGALS9 expression predicted decreased overall and progression-free survival in patients with lung cancer ("KM plot", http://kmplot.com/analysis/index.php?p=service&cancer=lung). (D) Kaplan Meier survival curves

by log-rank tests showed that a high mRNA level of LGALS9 in lung cancer tissue from the HKU cohort predicted poor overall survival, as determined by qPCR. (E) Correlation analysis between the tumor expression level of galectin-9 and the serum level of galectin-9 from the same lung cancer patients analyzed by Chi-square test and Peason correlation analysis. (F) Kaplan Meier survival curves by log-rank tests showed that serum galectin-9 level of lung cancer patients did not predict survival outcomes.

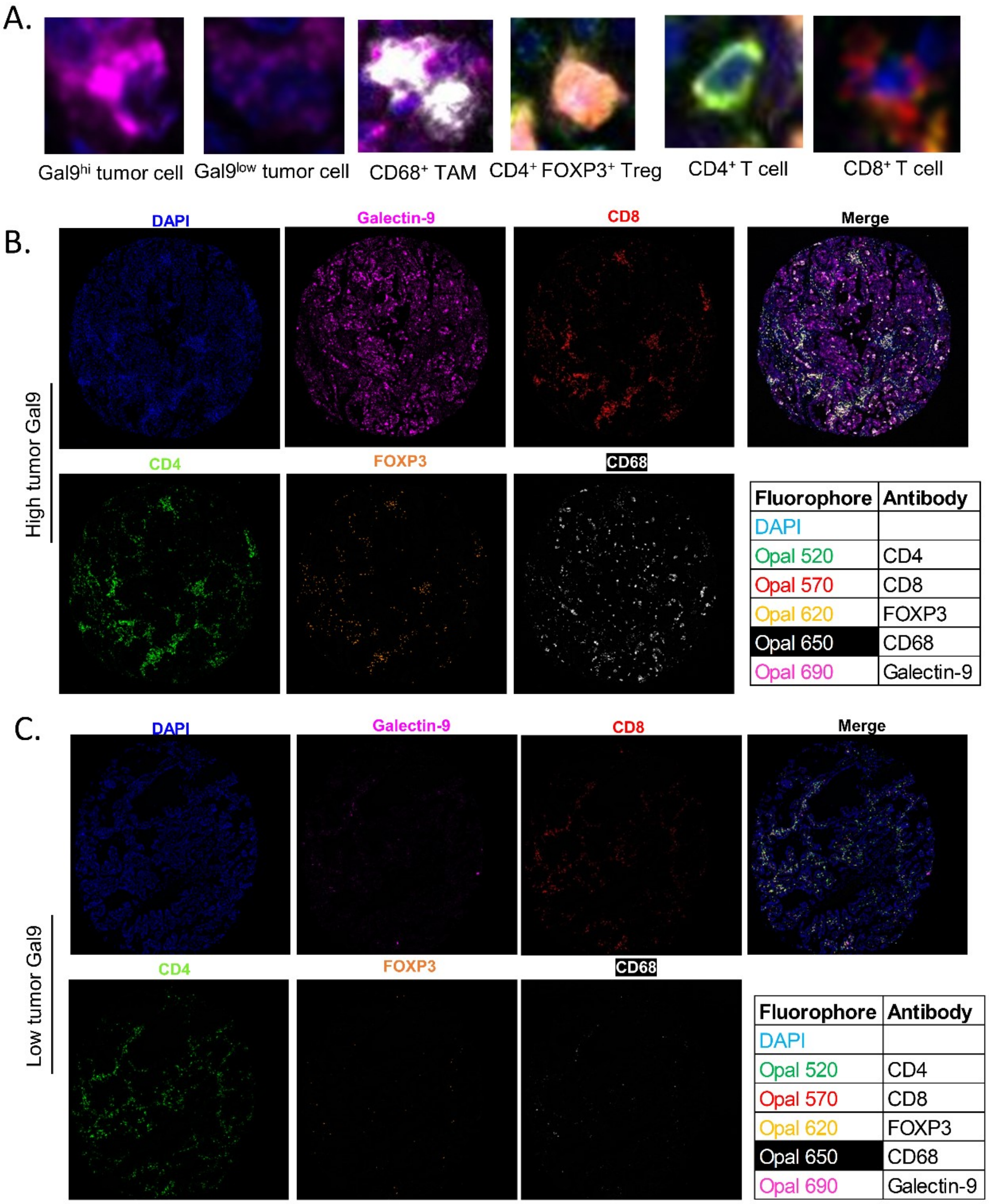

Figure S6. (A) The Galectin-9 expression pattern and correlation with immune cell infiltration were studied by performing 6-color multiplex fluorescent immunohistochemistry, and images were scanned and analyzed by the Vectra polaris system and Inform software. Representative images for cell phenotyping. Tumor cells were defined by the size of the nucleus indicated by the DAPI signal (blue), tumor-associated macrophages (TAMs) were identified by CD68 (white), Treg cells were

identified by coexpression of CD4 (green) and FOXP3 (orange), $CD4^+$ T cells were identified by CD4, and $CD8^+$ T cells were identified by CD8 (red). (B) Representative single-color images and merged images of DAPI, galectin-9, CD4, CD8, Foxp3 and CD68 staining of high tumor galectin-9 in the LUAD core. (C) Representative single color images and merged images of DAPI, galectin-9, CD4, CD8, Foxp3 and CD68 staining of low tumor galectin-9 LUAD core.

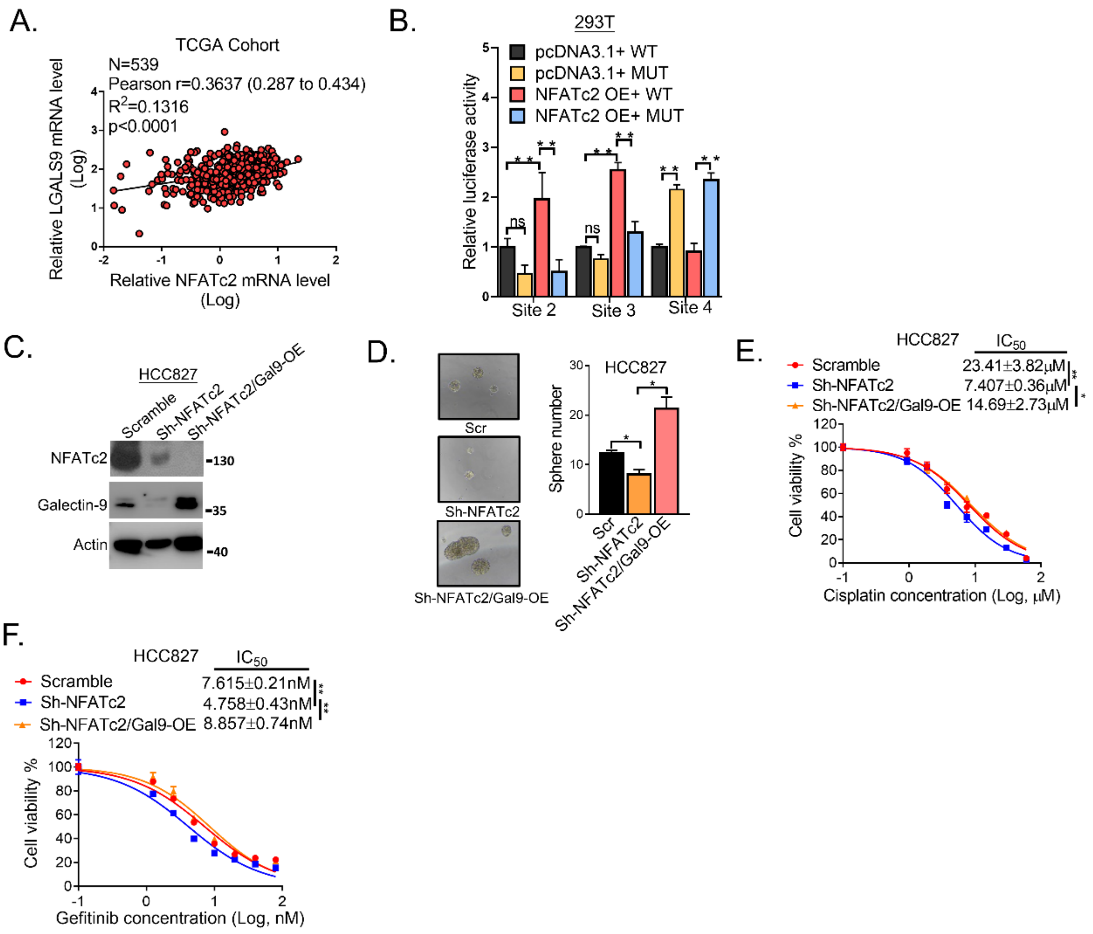

Figure S7. (A) Correlation of *LGALS9* and *NFATc2* mRNA expression levels determined by qPCR in a local human LUAD cohort analyzed by Pearson correlation analysis. (B) Luciferase activity of LGALS9 reporters with NFATc2-WT or NFATc2-MUT binding sites in 293T cells with or without NFATc2 overexpression determined by dual luciferase reporter assay. (C) Western blot analysis of the expression of galectin-9 and NFATc2 in HCC827 cells with or without NFATc2 and galectin-9 manipulation (D) Sphere formation assay performed in HCC827 cells with or without NFATc2/galectin-9 manipulation. (E-F) Cell viability assay of cisplatin (E) and gefitinib (F) performed on HCC827 cells with NFATc2/galectin-9 manipulation. *p<0.05; **p<0.01 compared with the control by one-way ANOVA corrected by Tukey's test. The data are presented as the means±SDs of three replicates.
